# HIF1 inhibitor acriflavine rescues early-onset preeclampsia phenotype in mice lacking placental prolyl hydroxylase domain protein 2

**DOI:** 10.1172/jci.insight.158908

**Published:** 2022-12-08

**Authors:** Julien Sallais, Chanho Park, Sruthi Alahari, Tyler Porter, Ruizhe Liu, Merve Kurt, Abby Farrell, Martin Post, Isabella Caniggia

**Affiliations:** 1 Lunenfeld-Tanenbaum Research Institute, Sinai Health System, Toronto, Ontario, Canada.; 2 Institute of Medical Sciences, and; 3 Department of Physiology, University of Toronto, Ontario, Canada.; 4 Program in Translational Medicine, Peter Gilgan Centre for Research and Learning, The Hospital for Sick Children, Toronto, Ontario, Canada.; 5 Department of Obstetrics & Gynaecology, University of Toronto, Ontario, Canada.

**Keywords:** Development, Reproductive Biology, Hypertension, Hypoxia, Mouse models

## Abstract

Preeclampsia is a serious pregnancy disorder that lacks effective treatments other than delivery. Improper sensing of oxygen changes during placentation by prolyl hydroxylases (PHDs), specifically PHD2, causes placental hypoxia-inducible factor-1 (HIF1) buildup and abnormal downstream signaling in early-onset preeclampsia, yet therapeutic targeting of HIF1 has never been attempted. Here we generated a conditional (placenta-specific) knockout of *Phd2* in mice (*Phd2^–/–^* cKO) to reproduce HIF1 excess and to assess anti-HIF therapy. Conditional deletion of *Phd2* in the junctional zone during pregnancy increased placental HIF1 content, resulting in abnormal placentation, impaired remodeling of the uterine spiral arteries, and fetal growth restriction. Pregnant dams developed new-onset hypertension at midgestation (E9.5) in addition to proteinuria and renal and cardiac pathology, hallmarks of severe preeclampsia in humans. Daily injection of acriflavine, a small molecule inhibitor of HIF1, to pregnant *Phd2^–/–^* cKO mice from E7.5 (prior to hypertension) or E10.5 (after hypertension had been established) to E14.5 corrected placental dysmorphologies and improved fetal growth. Moreover, it reduced maternal blood pressure and reverted renal and myocardial pathology. Thus, therapeutic targeting of the HIF pathway may improve placental development and function, as well as maternal and fetal health, in preeclampsia.

## Graphical abstract

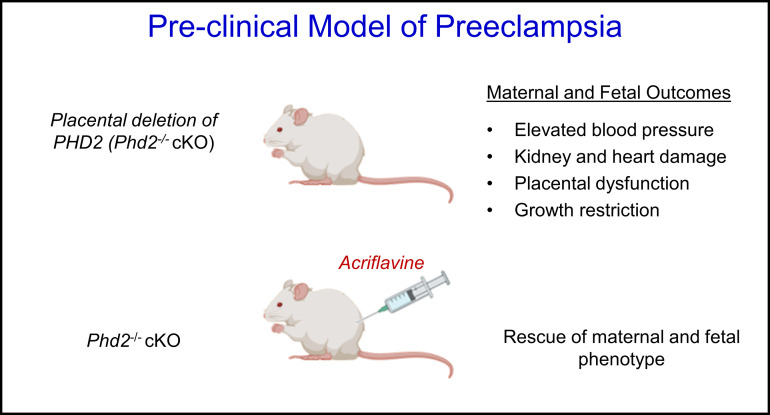

## Introduction

Preeclampsia is a common pregnancy disorder and leading cause of both maternal and fetal morbidity and mortality. It affects 2%–8% of all pregnancies worldwide, accounts for 10% of maternal mortality, and is the third most common cause of maternal death in North America. Preeclampsia is defined by new onset of hypertension (systolic blood pressure ≥ 140 mmHg and diastolic blood pressure ≥ 90 mmHg or in severe preeclampsia systolic blood pressure ≥ 160 mmHg, diastolic blood pressure ≥ 110 mmHg or above) in pregnant individuals often manifesting after 20 weeks of gestation and/or near term (1). Preeclampsia is a disorder often associated with proteinuria, or in the absence of it, with maternal organ dysfunction (such as but not limited to impaired liver function, renal insufficiency, and pulmonary edema) and fetal growth restriction (2). Severe preeclampsia may progress to eclampsia, the convulsive manifestation of gestational hypertension. Preeclampsia can manifest as early-onset preeclampsia (E-PE; symptoms arise ≤34 weeks gestation) or late-onset preeclampsia (L-PE; symptoms arise ≥34 weeks of gestation), with E-PE having more unfavorable maternal and fetal outcomes. E-PE and L-PE have distinct etiology and exhibit different molecular signatures (3, 4). E-PE is typically caused by a failure of the placenta (5) that adversely affects the uteroplacental circulation, culminating in chronic hypoxia. Secondary maternal clinical manifestations, largely due to excessive release of placental debris in the circulation, ending in a generalized maternal endothelial dysfunction, may also present as early as the second trimester of gestation. There is no cure apart from premature delivery of the placenta and fetus. To lessen the burden of disease, experimental animal models of E-PE are required to identify underlying pathophysiology and to develop and test new therapeutic compounds. Various experimental models have been developed; however, none has reproduced all aspects of E-PE (6, 7).

In humans, early placental development occurs in low oxygen that progressively rises during the first trimester of gestation (8, 9), and these changes in oxygen tension during this time tightly regulate placental trophoblast differentiation (10, 11). Hypoxia-inducible factor (HIF) is a master regulator of oxygen homeostasis and is essential for placental development (8, 10–12). Under low oxygen, the α subunit of HIF (HIF1A) accumulates in the nucleus, where upon binding to the HIF1B subunit, it recognizes HIF-responsive elements (HREs) within the promoter regions of hypoxia-responsive target genes (13, 14). Under normoxic conditions, HIF1A is rapidly degraded via a process that involves prolyl hydroxylase domain (PHD) proteins (15). In the presence of oxygen, PHD hydroxylates specific proline residues on HIF1A (15), resulting in their ubiquitination by von Hippel-Lindau tumor suppressor ubiquitin ligase that then provokes HIF1A proteasomal degradation (16). Although all 3 isoforms of PHD (PHD1–3) are expressed in the human placenta, PHD2 is the primary regulator of HIF1A and the only isoform reduced in E-PE (3, 8, 17). Gene deletions of *Phd1–3* in mice revealed that only *Phd2*-deficient mice die early in utero due to placental defects (18), highlighting the importance of PHD2 for placenta development. However, occurrence of preeclampsia in these global *Phd2*-deficient mice was not determined.

E-PE is hallmarked by elevated HIF1A levels (10, 19). Genetic and pharmacological manipulations causing increased HIF1A signaling in pregnant mice have indeed led to complications resembling preeclampsia (20–23). However, excess of HIF1A in other organs than placenta and associated fetal/maternal morbidities are confounding these murine models of preeclampsia. Trophoblastic overexpression of constitutively active HIF1A in mice reproduces preeclampsia-like symptoms, although fetal weight was only reduced at birth (24), contrasting early growth restriction in human E-PE. Previously, we have reported that heightened HIF1A levels in human E-PE, but not L-PE, placentae (10) are a consequence of aberrant oxygen sensing due to diminished placental PHD2 expression and function (3). This, in turn, contributes to decreased HIF1A hydroxylation and degradation, leading to its overexpression in E-PE placenta and, consequently, to altered placental development (3). We reasoned that a placenta-specific *Phd2* deletion in mice would better recapitulate the early-onset spectrum of preeclampsia than broad trophoblastic overexpression of constitutively active HIF1A (24). Here we report that loss of *Phd2* in the junctional zone of the murine placenta leads to an increase in HIF1A and an E-PE phenotype. In contrast to mice with global *Phd2* deficiency (18), the placental (conditional) *Phd2* knockout (*Phd2^–/–^* cKO) is not embryonically lethal, making it a suitable preeclampsia model for examining anti-HIF-1A therapy. We show that administration of acriflavine, a small molecule inhibitor of HIF1 (25, 26), to pregnant *Phd2^–/–^* cKO mice ameliorated the maternal, fetal, and placental preeclampsia-like features.

## Results

### Placental deletion of PHD2 alters placental architecture and impairs spiral artery remodeling.

**Figure 1:**
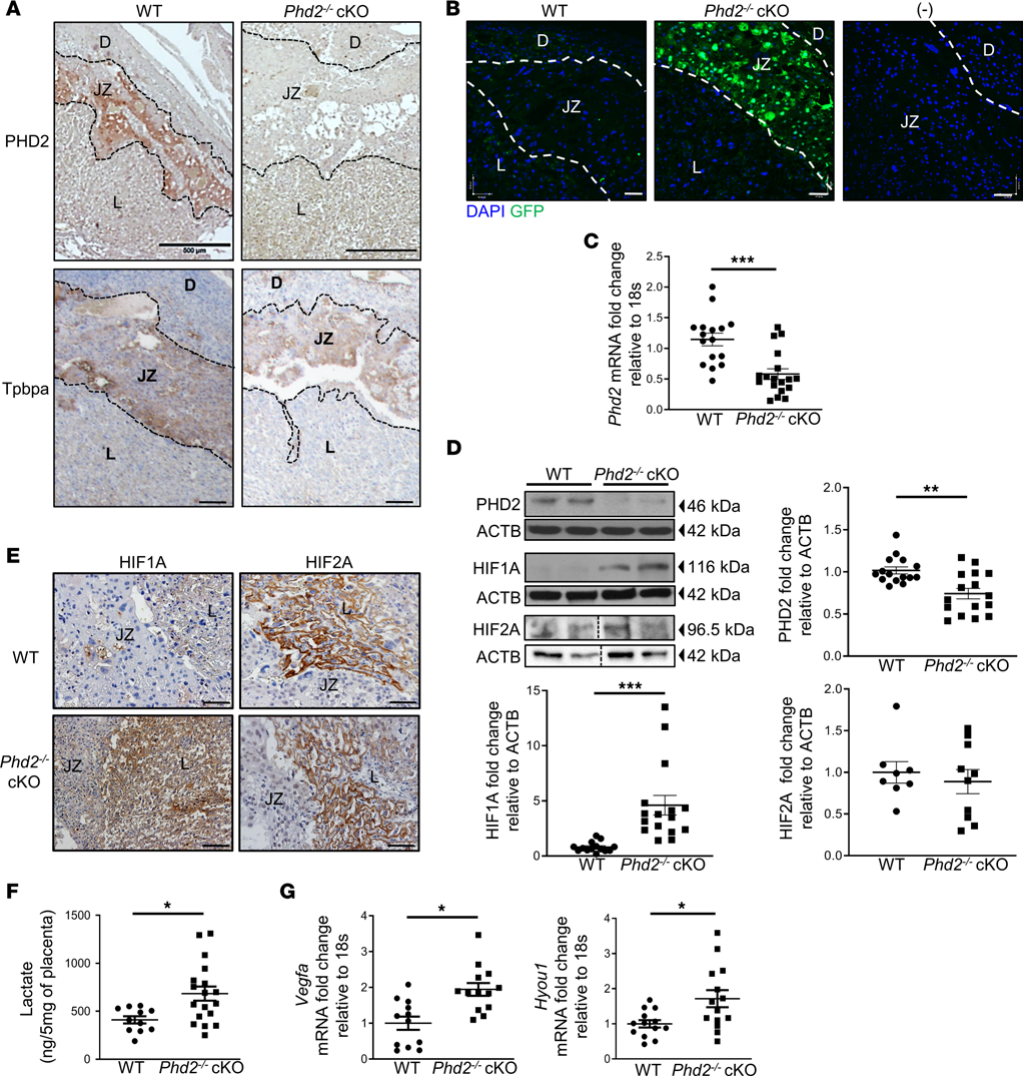
Deletion of *Phd2* in JZ layer increases placental HIF1A content and activity. (**A**) IHC staining for PHD2 (top panel, scale bars = 500 μm) and Tpbpa (bottom panel, scale bars = 100 μm) in WT and *Phd2^–/–^* cKO placental sections at E14.5 (*n* = 3 WT and *Phd2^–/–^* cKO placentae, respectively). (**B**) Immunofluorescence (IF) staining for GFP (marks Cre recombinase expression) in sections from WT and *Phd2^–/–^* cKO placentae at E14.5 (*n* = 3 WT and *Phd2^–/–^* cKO placentae respectively; all scale bars represent 45 μm). (**C**) Quantitative PCR (qPCR) for Phd2 in E14.5 WT and *Phd2^–/–^* cKO placentae (Phd2, ***P < 0.001 relative to WT, unpaired Student’s *t* test, *n* = 16 WT, *n* = 18 *Phd2^–/–^* cKO placentae). (**D**) Representative Western blot (WB) for PHD2, HIF1A, and HIF2A and associated densitometry in whole placental lysates from WT and *Phd2^–/–^* cKO placentae (PHD2, **P < 0.01 relative to WT, unpaired Student’s *t* test, *n* = 15 WT and *n* = 16 *Phd2^–/–^* cKO placentae; HIF1A, ***P < 0.001 relative to WT, unpaired Student’s *t* test, *n* = 14 WT and *n* = 16 *Phd2^–/–^* cKO placentae; HIF2A, no significant difference relative to WT, *n* = 8 WT and *n* = 10 *Phd2^–/–^* cKO placentae; bottom panel HIF2A and ACTB lanes were run on the same gel but were noncontiguous). (**E**) IHC staining for HIF1A and HIF2A in placenta sections from WT and *Phd2^–/–^* cKO placentae at E14.5 (*n* = 3 WT and *Phd2^–/–^* cKO placentae respectively, scale bar = 50 μm). (**F**) Lactate content measured by tandem mass spectrometry in E14.5 WT and *Phd2^–/–^* cKO placentae (*P < 0.05 compared to WT, unpaired Student’s *t* test, *n* =11 WT and *n* = 18 *Phd2^–/–^* cKO placentae). (**G**) qPCR for *Vegfa* and *Hyou1* in E14.5 WT and *Phd2^–/–^* cKO placentae (*P < 0.05 compared with WT, unpaired Student’s *t* test; *Vegfa*, *n* = 12 WT and *n* = 13 *Phd2^–/–^* cKO placentae; *Hyou1*, *n* = 13 WT and *n* = 14 *Phd2^–/–^* cKO placentae). Data are expressed as fold change, relative to the 18S. D, decidua; JZ, junctional zone; L, labyrinth; WT, wild-type; cKO, conditional knockout.

Immunohistochemical (IHC) staining for PHD2 revealed prominent PHD2 protein expression in the junctional zone (JZ) of E14.5 mouse placenta (Figure 1A). To delete PHD2 expression in the JZ layer, we crossed *Phd2^fl/fl^* mice (27) with homozygous *4311^cre-EGFP^* (*Tpbpa^cre-EGFP^*) transgenic mice for successive rounds to obtain homozygous *Tpbpa^cre^ Phd2^fl/fl^* mice. Tpbpa-cre mice express Cre recombinase in the spongiotrophoblast cells of the JZ, the layer that gives rise to trophoblast giant cells and glycogen cells (28). Homozygous *Tpbpa^cre^ Phd2^fl/fl^* males were mated with female *Phd2^fl/fl^* mice to generate JZ*-*specific *Phd2^–/–^* cKO (*Tpbpa^cre^ Phd2^–/–^*) embryos whereas male *Phd2^fl/fl^* mice that after crossing lacked *Tpbpa^cre^* were bred with female *Phd2^fl/fl^* to produce control WT embryos. No issues with fertility were noted. Litter size at E14.5 was reduced in pregnant *Phd2*^–/–^ cKO mice compared with pregnant WT mice (Supplemental Table 1; supplemental material available online with this article; https://doi.org/10.1172/jci.insight.158908DS1). However, more than 80% of offspring survived, and the sex ratio was not affected. Pregnant mice were sacrificed at E14.5 and 17.5 (1 day prior to birth) to allow for placental and maternal phenotypic analysis.

Immunohistochemistry for PHD2 showed absence of PHD2 protein in the JZ layer of E14.5 placental sections of *Phd2^–/–^* cKO embryos (Figure 1A). IF for GFP confirmed localized expression of Cre recombinase in the JZ of the *Phd2^–/–^* cKO placenta (Figure 1B) (28). Tpbpa protein content in the JZ was unchanged (Figure 1A). Real-time PCR and immunoblotting confirmed reduced PHD2 mRNA (Figure 1C) and protein (Figure 1D and see complete unedited blots in the supplemental material) expression in E14.5 whole placentae of *Phd2^–/–^* cKO embryos. Diminished placental PHD2 expression in *Phd2^–/–^* cKO embryos was accompanied by an increase in HIF1A, but not HIF2A, protein (Figure 1D and full, uncut gels). IHC revealed increased HIF1A immunoreactivity in both JZ and labyrinth layer of *Phd2^–/–^* cKO compared with WT placentae (Figure 1E). In contrast, HIF2A immunoreactivity did not change and was confined to the labyrinth layer of both WT and *Phd2^–/–^* cKO placentae (Figure 1E). Hypoxic status of *Phd2^–/–^* cKO placenta was corroborated by detection of pimonidazole adducts in both JZ and labyrinth layer following hypoxyprobe staining (29) (Supplemental Figure 1A). Lactate content, an indicator of increased HIF1-mediated glycolysis (30), and expression (31) of HIF1 target genes vascular endothelial growth factor A (*Vegfa*) and hypoxia upregulated 1 (*Hyou1*) (32, 33) were significantly increased in E14.5 placentae of *Phd2^–/–^* cKO embryos relative to WT controls (Figure 1, F and G). Thus, the JZ-specific deletion of *Phd2* was effective in upregulating HIF1-mediated activities in the placenta.

**Figure 2:**
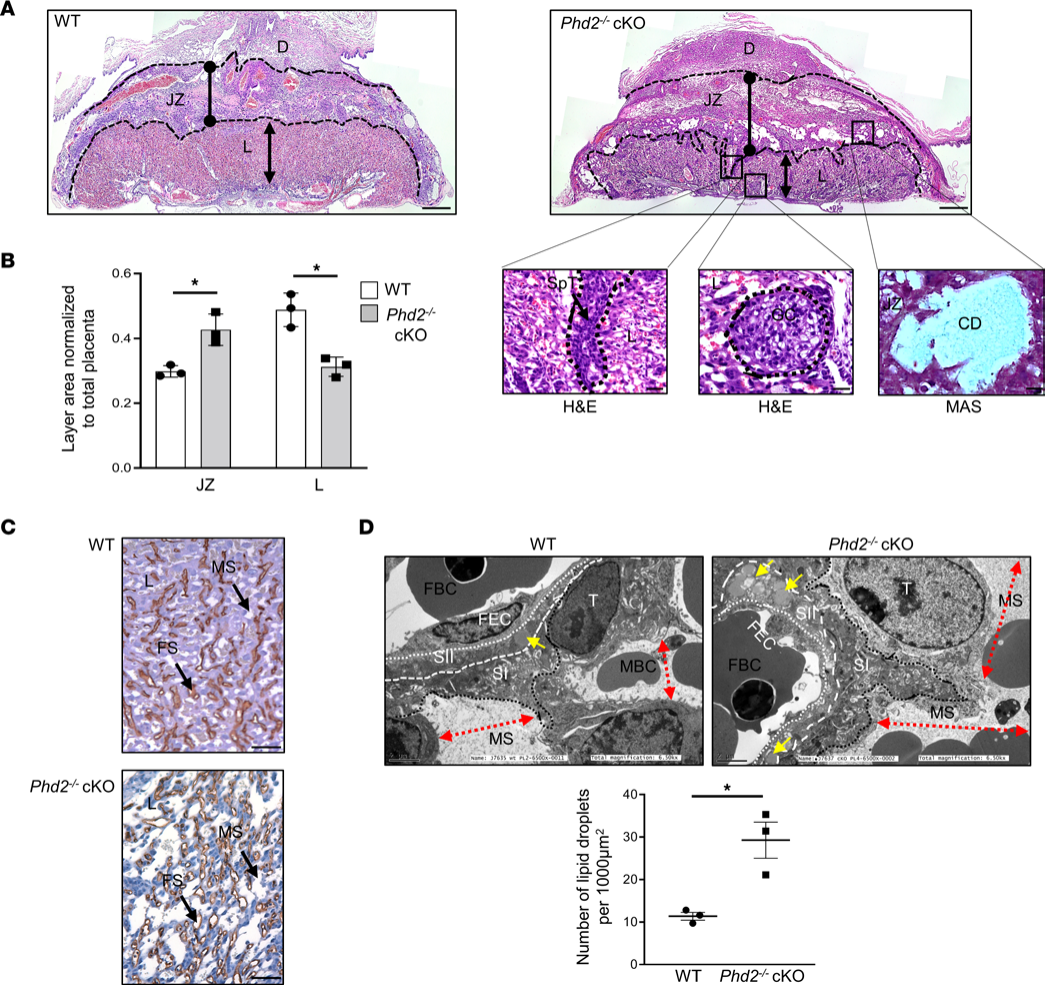
Removal of *Phd2* in JZ layer alters placental architecture. (**A**) H&E staining of WT (left) and *Phd2*^–/–^ cKO (right) placentae at gestational day 14.5 (scale bars represent 100 μm). Higher magnifications (scale bars represent 25 μm) show invagination of spongiotrophoblast cells (left insert) and mislocalization of JZ-restricted glycogen cells (middle insert) in the labyrinth layer while MAS staining (right insert) revealed collagen deposits in the JZ layer of *Phd2*^–/–^ cKO placentae (*n* = 4 WT and *Phd2^–/–^* cKO placentae respectively). (**B**) Morphometric analysis of placental layers (measured by delineating the total area of each layer) of E14.5 WT and *Phd2*^–/–^ cKO placentae. Size of labyrinth (L) and junctional zone (JZ) are expressed as percentage of whole placenta (*P < 0.05, 1-way ANOVA, Tukey posttest; *n* = 4 WT and *n* = 4 *Phd2*^–/–^ cKO placentae). (**C**) Representative IHC staining for CD34 (endothelial cell marker) in E14.5 WT and *Phd2*^–/–^ cKO placentae (*n* = 4 WT and *Phd2^–/–^* cKO placentae respectively, scale bars represent 50 μm). (**D**) Representative transmission electron microscopy (TEM) images of the labyrinth of E14.5 WT and *Phd2*^–/–^ cKO placentae and associated lipid droplet count (*P < 0.05, unpaired Student’s *t* test; TEM images/placenta; *n* = 3 WT and *n* = 3 *Phd2*^–/–^ cKO placentae). Yellow arrowheads indicate lipid droplets in SII, while red double arrow lines indicate size of maternal sinusoids. D, decidua; FS, fetal sinusoids; GC, glycogen cells; CD, collagen deposits; FBC, fetal blood cell; FEC, fetal endothelial cell; JZ, junctional zone; L, labyrinth; MAS, Masson’s trichrome stain; MS, maternal sinusoids; MBC, maternal blood cells; T, trophoblast cell; SI, syncytiotrophoblast layer I; SII, syncytiotrophoblast layer II; SpT, spongiotrophoblast.

While no differences in placental weight were found at 14.5 and 17.5 days of gestation between WT and *Phd2^–/–^* cKO embryos (Supplemental Figure 1B), histological examination of E14.5 placental sections revealed striking structural alterations (Figure 2A). We noted significant compaction of the labyrinth and expansion of the JZ in *Phd2^–/–^* cKO versus WT placentae (Figure 2, A and B). This was accompanied by SpT invaginations and glycogen cells being mislocalized in the labyrinth (Figure 2A, enlarged insets). The JZ of the *Phd2^–/–^* cKO placentae had more SpT and glycogen cells as manifest by Tpbpa-positive signal (Supplemental Figure 2, A and B) and contained fibrotic collagen deposits (Figure 2A, enlarged insets). IHC for CD34, marker of endothelial cells, revealed a less complex vascularity and dilation of maternal sinusoids in *Phd2^–/–^* cKO placentae (Figure 2C) that was corroborated at the ultrastructural level (Figure 2D), implying alterations in fetoplacental vascularity (34). Additionally, TEM revealed a significant accumulation of lipid droplets in the syncytiotrophoblast layer II (Figure 2D), suggestive of diminished placental lipid trafficking to the developing fetus (31).

**Figure 3:**
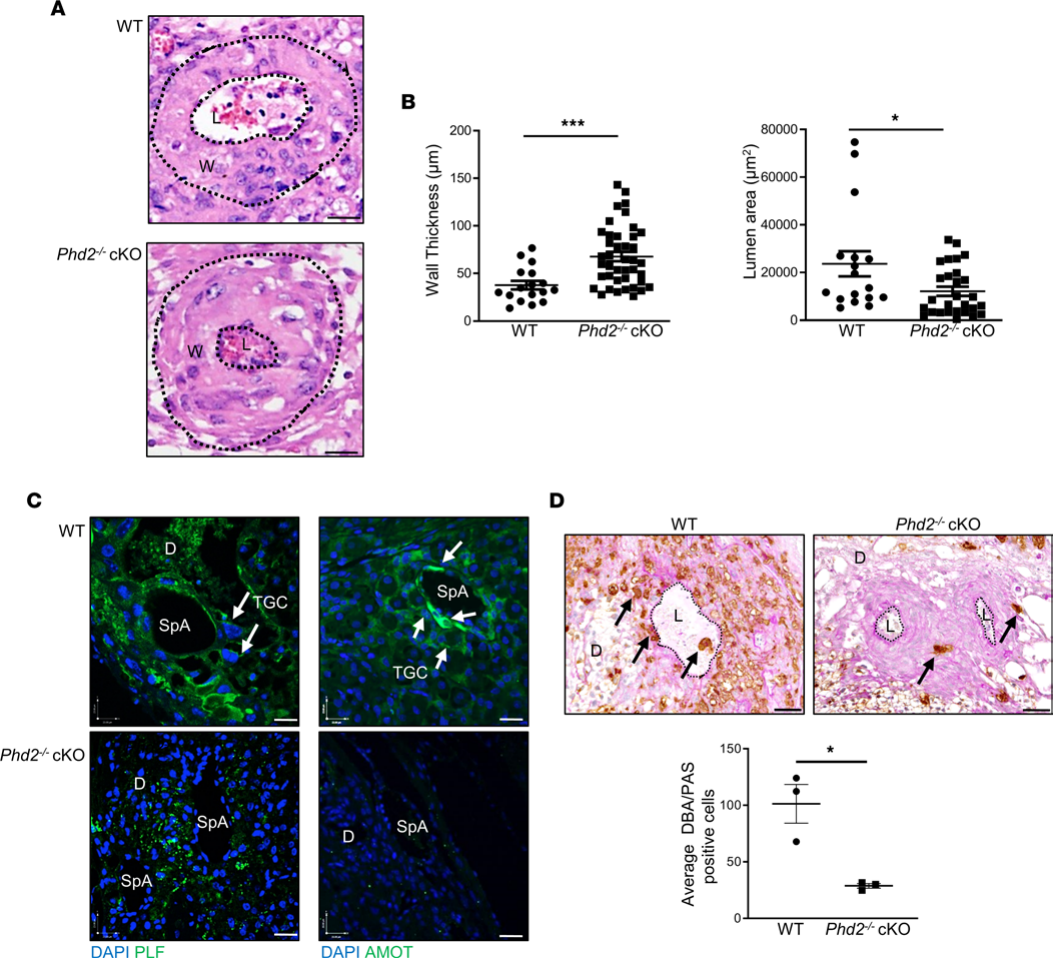
Deletion of *Phd2* in JZ layer impairs decidual SpA remodeling. (**A**) Representative H&E staining of decidual SpAs in WT and *Phd2*^–/–^ cKO placental beds at gestational day 14.5 (scale bars represent 25 μm). Outer and inner wall of the arteries are delineated by dotted lines. W, wall; L, lumen. (**B**) Wall thickness and lumen area measurements of decidual SpAs in WT and *Phd2*^–/–^ cKO placentae (*P < 0.05, ***P < 0.001 relative to the WT, unpaired Student’s *t* test, *n* = 17 SpAs of 6 WT placentae and *n* = 31 SpAs of 12 *Phd2*^–/–^ cKO placentae). (**C**) IF staining for proliferin (PLF) and angiomotin (AMOT) of E14.5 WT and *Phd2*^–/–^ cKO placental beds; nuclei were visualized with DAPI (all scale bars represent 25 μm). D, decidua; TGC, trophoblast giant cells; arrow, immunopositive TGCs. (**D**) Representative PAS/DBA double-staining (arrows) and associated count for uNK cells (DBA/PAS-positive cells. *P < 0.05; unpaired Student’s *t* test; data represent average of 4 images/placental bed; *n* = 3 WT and *Phd2*^–/–^ cKO) surrounding SpAs of E14.5 WT and *Phd2*^–/–^ cKO placentae (scale bars represent 50 μm). D, decidua; SpA, SpA; L, lumen.

During murine placentation, trophoblast giant cells (TGCs) invade the endometrial stroma and displace endothelial cells of maternal spiral arteries (SpAs). This transforms these vessels into high-caliber conduits that funnel maternal blood to the placenta, thereby optimizing gas and nutrient exchange from the mother to the developing fetus (35). Defective remodeling of uterine SpAs is one of the hallmarks of preeclampsia in humans (36). Therefore, we assessed SpA remodeling in our pregnant *Phd2^–/–^* cKO mice at E14.5 using H&E-stained sections of the central part of the decidua basalis. Decidual SpAs of the *Phd2*^–/–^ cKO placental bed had significantly smaller lumen area and increased wall thickness than those of the WT placental bed (Figure 3, A and B). Immunofluorescence (IF) staining for proliferin (PLF), and angiomotin (AMOT), markers for invading spiral artery–associated trophoblast giant cells (SpA-TGCs) (37, 38), verified impaired TGC invasion of maternal SpA vessels (Figure 3C). While ample SpA-TGCs near and within the SpAs were identified in the WT placental bed, only a few were present in proximity of SpAs of the *Phd2^–/–^* cKO placental bed (Figure 3C). As uterine natural killer (uNK) cells have been implicated in SpA remodeling (39, 40), we double-stained placental sections with periodic acid–Schiff (PAS) and Dolichos biflorus agglutinin (DBA) to visualize uNK cells. We observed a significant decrease in double-positive uNK cells around the SpAs of the *Phd2^–/–^* cKO compared with WT placental bed (Figure 3D), in line with reported reduction of SpA-associated uNK cells in human preeclampsia (41–43). However, other reports have shown no change (44) or an increase (45) in uNK cells in preeclamptic women. Reduced presence of uNK cells in proximity to SpAs in *Phd2^–/–^* cKO placental bed was supported by staining for CD69 (Supplemental Figure 2C), a marker of decidual uNK cells (46).

### Loss of placental PHD2 impairs fetal growth and provokes maternal symptoms of preeclampsia.

**Figure 4:**
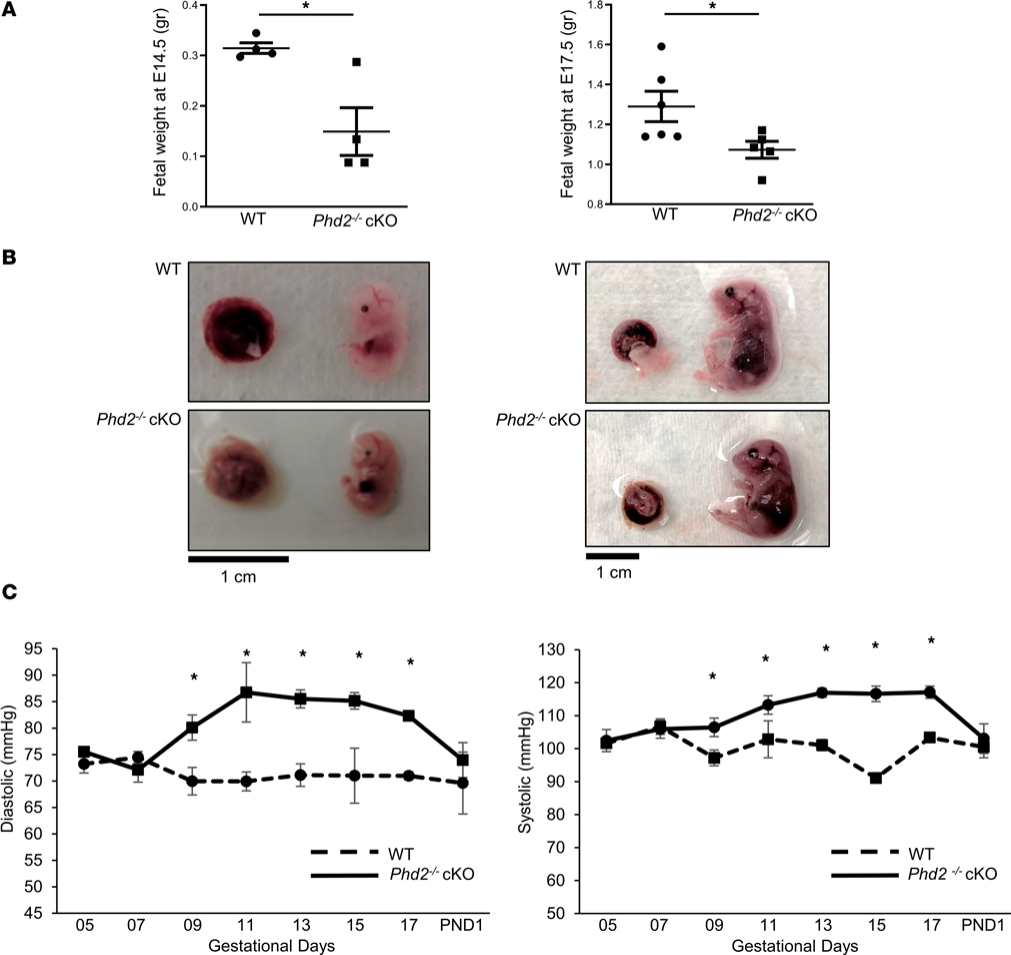
Loss of *Phd2* in JZ layer results in fetal growth restriction and elevated maternal blood pressure. (**A**) Average weight of embryos from WT and *Phd2^–/–^* cKO pregnant mothers immediately after sacrifice at gestational day 14.5 (*P < 0.05 relative to the WT, unpaired Student’s *t* test; *n* = 4 WT litters, 38 embryos, and *n* = 4 *Phd2*^–/–^ cKO litters, 37 embryos) and gestational day 17.5 (*P < 0.05 relative to the WT, unpaired Student’s *t* test; *n* = 6 WT litters, 71 embryos, and *n* = 5 *Phd2*^–/–^ cKO litters, 52 embryos). (**B**) Representative gross morphology of placentae and embryos of WT and *Phd2^–/–^* cKO pregnant mice taken after sacrifice at gestational day 14.5 and 17.5 (scale bars represent 1 cm). (**C**) Maternal diastolic and systolic pressures across gestation in WT and *Phd2*^–/–^ cKO mice (*P < 0.05 relative to the WT at each matching gestational day, unpaired Student’s *t* test; *n* ≥ 5 separate pregnant mothers per condition).

In humans, impaired SpA remodeling is known to associate with placental hypoxia and reduced fetal growth (5). *Phd2^–/–^* cKO embryos, at both E14.5 and 17.5, displayed reduced fetal weight in utero compared with WT (*Phd2^fl/fl^*) embryos, corroborating impaired placental efficiency (Figure 4, A and B). Since preeclampsia is primarily diagnosed by new onset of maternal hypertension, we monitored daily the evolution of maternal blood pressure in our *Phd2^–/–^* cKO and WT (*Phd2^fl/fl^*) pregnant mothers. Pregnant WT mice displayed constant blood pressure throughout pregnancy, being about 70 mmHg for diastolic and about 100 mmHg for systolic pressures (Figure 4C). *Phd2^–/–^* cKO pregnant mice exhibited elevated blood pressure compared with WT pregnant mice with diastolic and systolic values averaging 85 and 115 mmHg, respectively (Figure 4C). The rise in maternal blood pressure started around E9.5 (Figure 4C), coinciding with peak TGC invasion and remodeling of the SpAs (35). We allowed a set of *Phd2^–/–^* cKO mothers to deliver and found that their blood pressure returned to normal values 1 day after delivery (Figure 4C). These data indicate that placental loss of PHD2 in pregnant mice provokes new-onset maternal hypertension that resolves after delivery, like that seen in human early-onset preeclampsia.

**Figure 5:**
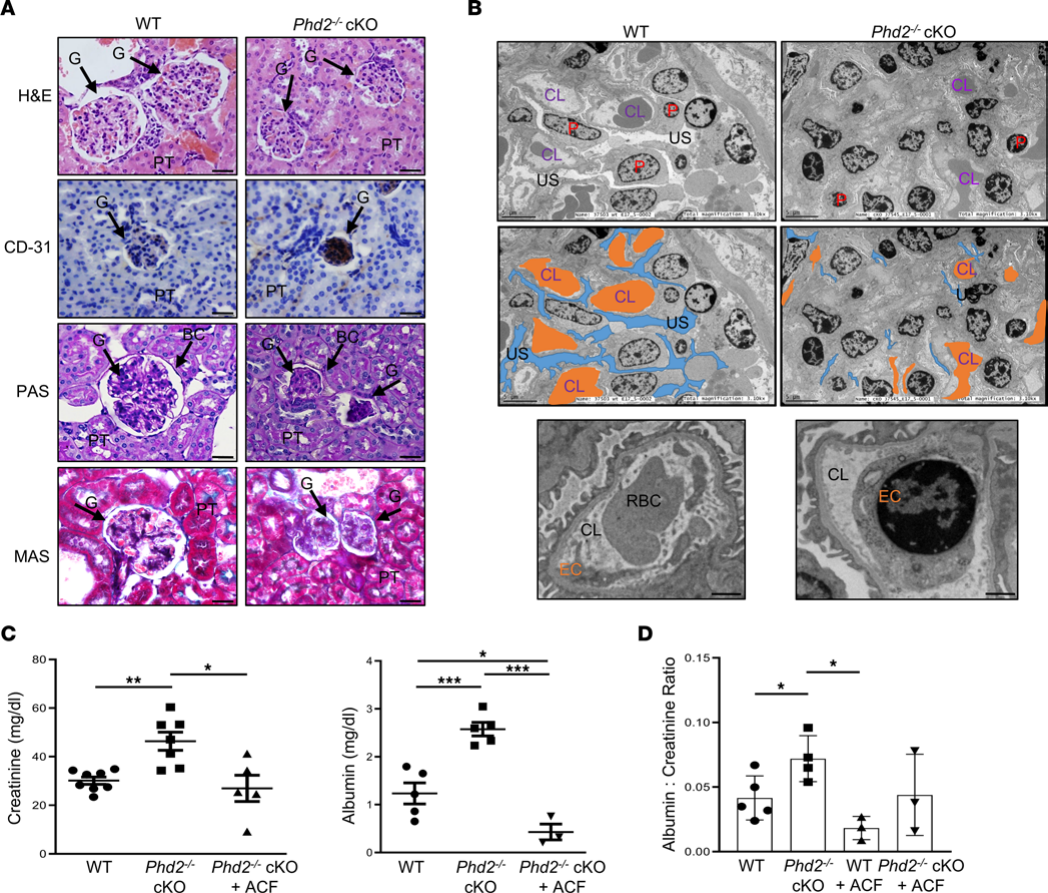
Removal of *Phd2* in JZ layer leads to glomerular endotheliosis in the mother. (**A**) Representative H&E, PAS, and MAS staining and IHC for CD31 of sagittal maternal kidney sections at day 17.5 of pregnancy from WT and *Phd2*^–/–^ cKO pregnant mice. G, glomeruli; PT, proximal tubules; BC, Bowman’s capsule (arrows indicate location of the glomeruli; scale bars represent 25 μm). (**B**) Representative TEM images of glomeruli from maternal kidneys of WT and *Phd2*^–/–^ cKO pregnant mice at day 17.5 of pregnancy. US, urinary spaces; CL, capillary loop; P, podocytes; EC, endothelial cell (top panel: scale bars represent 5 μm; bottom panel: scale bars represent 1 μm). (**C**) Creatinine and albumin content and (**D**) albumin/creatinine ratio in urine obtained at gestational day 17.5 from WT and *Phd2*^–/–^ cKO pregnant mice treated with either PBS or acriflavine (ACF) (*P < 0.05, **P < 0.01, ***P < 0.001, 1-way ANOVA, Newman-Keuls post hoc test, *n* = 8 separate WT pregnant dams, *n* = 7 separate *Phd2*^–/–^ cKO pregnant dams treated with PBS, *n* = 5 separate *Phd2*^–/–^ cKO pregnant dams treated with ACF).

The most severe form of preeclampsia is frequently associated with renal insufficiency, characterized by the development of glomerular endotheliosis that results in impaired permeability of glomerular capillaries and consequently proteinuria (47). Glomerular endotheliosis is characterized by a swelling of the endothelial cells and reduction of the capillary lumens (48). H&E staining of maternal kidney sections at day 17.5 of pregnancy demonstrated glomerular damage in *Phd2^–/–^* cKO compared with WT (*Phd2^fl/fl^*) pregnant mice (Figure 5A). CD31 staining revealed increased endothelial cell density in the glomeruli of *Phd2^–/–^* cKO compared with WT (*Phd2^fl/fl^*) pregnant mice (Figure 5A and Supplemental Figure 3A, bottom panels). In addition to heightened endothelial cell concentration, PAS staining revealed disrupted glomerular basement membranes and increased (dark purple) mesangial matrix in the glomeruli while MAS staining demonstrated a higher amount of fibril deposition (Figure 5A). Morphometric analyses corroborated these observations by demonstrating reduced glomerulus area and diameter as well as diminished Bowman space in *Phd2^–/–^* mice compared with WT (*Phd2^fl/fl^*) pregnant mice (Supplemental Figure 3B). These morphological alterations of glomeruli in the maternal kidney of *Phd2^–/–^* cKO pregnant mothers were supported by TEM (Figure 5B). Urinary space (normalized to total glomeruli area) and capillary lumens were significantly reduced in maternal kidneys of *Phd2^–/–^* cKO compared with WT (*Phd2^fl/fl^*) pregnant mice, and this associated with presence of enlarged endothelial cells (Figure 5B). These structural changes of the maternal kidneys were associated with elevated levels of creatinine (46.4 ± 3.73 versus 28.32 ± 1.47 mg/dL, *Phd2^–/–^* cKO versus WT, mean ± SEM) and albumin (1.24 ± 0.22 versus 2.58 ± 0.16 mg/dL, *Phd2^–/–^* cKO versus WT, mean ± SEM), as well as an increased albumin/creatinine ratio in the urine of *Phd2^–/–^* cKO pregnant mice (Figure 5, C and D).

**Figure 6:**
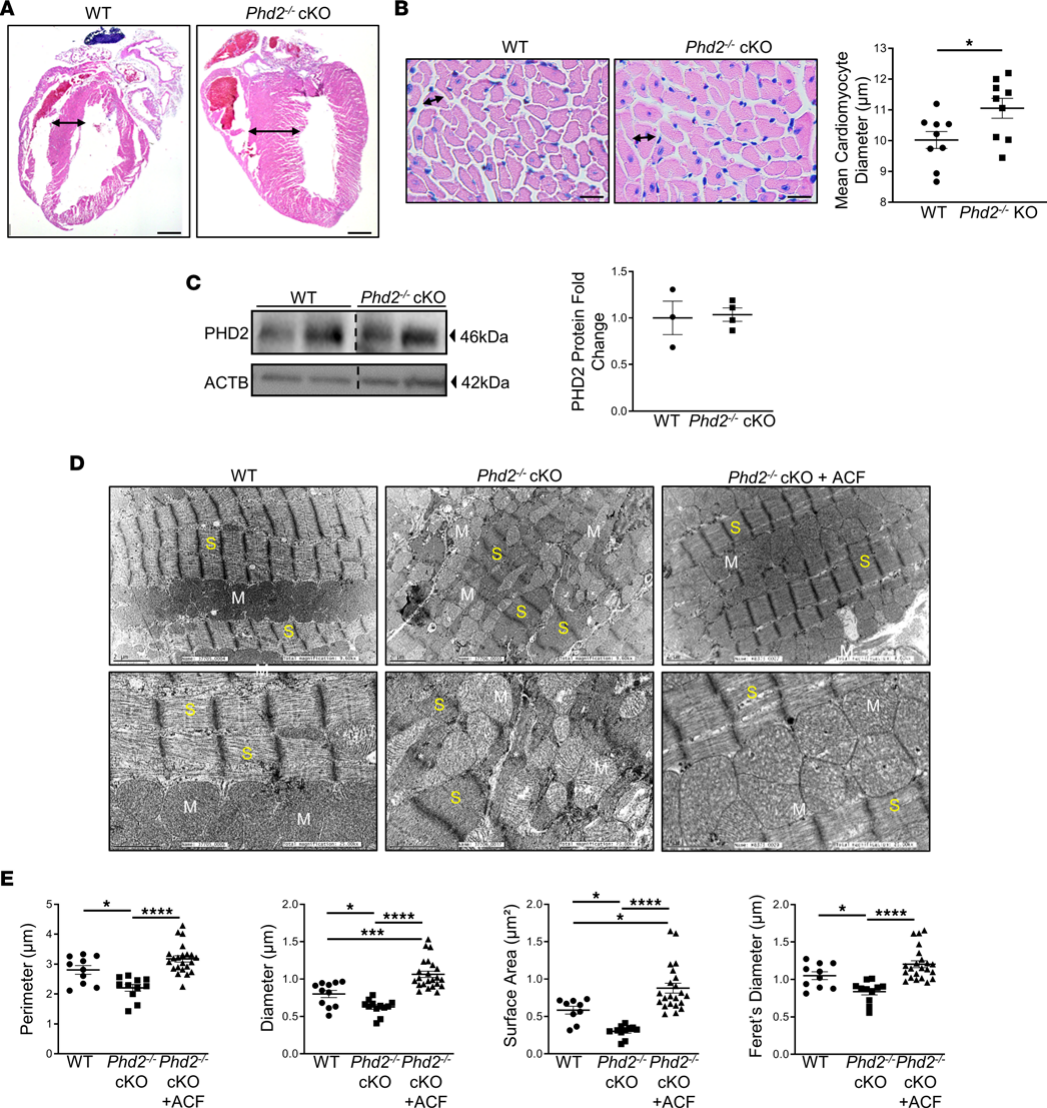
Placental removal of *Phd2* leads to maternal heart alterations. (**A**) Representative H&E staining of sagittal maternal heart sections at day 17.5 of pregnancy from WT and *Phd2*^–/–^ cKO pregnant mice (scale bars represent 1,000 μm). (**B**) Morphometric assessment of cardiomyocyte diameter in heart sections from WT and *Phd2*^–/–^ cKO mice at day 17.5 of pregnancy (*P < 0.05; unpaired Student’s *t* test; mean of 3 H&E images from LV wall per mouse; *n* = 3 WT and *n* = 3 *Phd2^–/–^* cKO dams, respectively; scale bars represent 20 μm). (**C**) Representative WBs for PHD2 and associated densitometry in whole heart lysates from WT and *Phd2^–/–^* cKO dams. Data are expressed as fold change relative to ACTB (no significant difference relative to WT, *n* = 3 WT and *n* = 4 *Phd2^–/–^* cKO placentae; PHD2 and ACTB lanes were run on the same gel but were noncontiguous). (**D**) Representative TEM images of maternal heart muscle at day 17.5 of gestation of WT, *Phd2*^–/–^ cKO and *Phd2*^–/–^ cKO mice injected with ACF during early pregnancy (GD7.5–14.5; top panel: scale bars represent 2 μm; bottom panel: scale bars represent 1 μm). White stars indicate fragmented mitochondria. M, mitochondria; S, sarcomere. (**E**) Mitochondrial morphometric analysis of cardiomyocytes at day 17.5 of gestation of WT, *Phd2*^–/–^ cKO, and *Phd2*^–/–^ cKO mice injected with ACF during early (GD7.5–14.5) pregnancy “(*P < 0.05, ***P < 0.001, ****P < 0.0001 relative to mitochondria of WT pregnant mice, 1-way ANOVA, Newman-Keuls post hoc test; minimum of 4 images per section; *n* = 3 separate pregnant mice per condition).

In women with early-onset preeclampsia, impaired placentation is associated with cardiac abnormalities including left ventricular hypertrophy and dysfunction (49). Histological staining of sagittal maternal heart sections day 17.5 of pregnancy demonstrated marked thickening of the left ventricular (LV) walls of *Phd2^–/–^* cKO versus WT pregnant dams (Figure 6A). Morphometric analysis of heart sections from LV walls showed a marked increased in cardiomyocyte diameter (Figure 6B), indicative of hypertrophy. No change in PHD2 protein content was found between heart lysates from WT and Phd2^–/–^ cKO pregnant dams (Figure 6C and full, uncut gels), suggesting that cardiac phenotype was not due to unintended leaky cre expression in cardiac cells. At the ultrastructural level the mitochondrial arrangement in maternal cardiomyocytes of *Phd2^–/–^* cKO pregnant dams was significantly altered compared with that of WT pregnant mothers (Figure 6D). In WT pregnant controls, mitochondria of maternal cardiomyocytes were aligned in longitudinal rows between the myofibrils and had the same length as a sarcomere whereas in *Phd2^–/–^* cKO pregnant dams these interfibrillar mitochondria were disorganized and exhibited different morphology. The mitochondria were smaller (decreased surface area and perimeter) and more fragmented (decreased Feret’s diameter and increase in the number of mitochondria per image) (Figure 6E), in line with a shift in morphological dynamics of mitochondria to fission (50). As preeclamptic women have an increased risk of developing heart failure later in life (51), it is plausible that these maternal cardiac dysmorphologies during pregnancy contribute to later heart disease.

### The small molecule HIF inhibitor acriflavine partially rescues the preeclamptic phenotype.

**Figure 7:**
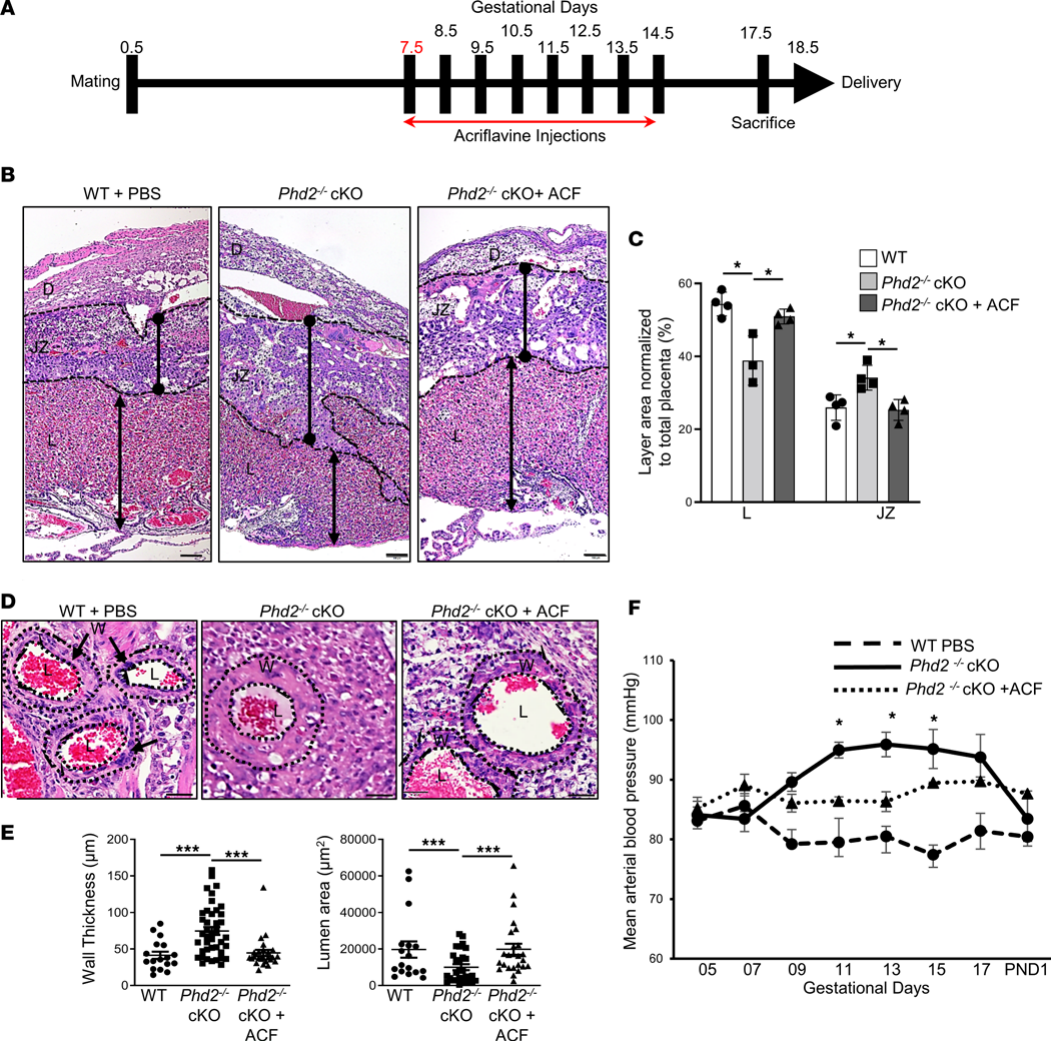
ACF administration in early pregnancy prevents placental, fetal, and maternal preeclamptic phenotype in JZ-specific *Phd2^–/–^* cKO pregnant mice. (**A**) Schematic of ACF injection regimen (GD7.5–14.5) during early pregnancy. (**B**) H&E staining of E17.5 placental sections from WT and *Phd2*^–/–^ cKO pregnant mice treated with either PBS or ACF (scale bars represent 100 μm). (**C**) Morphometric analysis of E17.5 placentae from WT and *Phd2*^–/–^ cKO pregnant mice treated with either PBS or ACF. Size of total labyrinth (L) and junctional zone (JZ) areas are expressed as a percentage of whole placenta (*P < 0.05, 1-way ANOVA, Newman-Keuls post hoc test; *n* = 9 WT placentae, *n* = 9 *Phd2*^–/–^ cKO placentae, *n* = 9 *Phd2*^–/–^ cKO placentae of ACF-treated pregnant dams). D, decidua. (**D**) H&E staining of decidual maternal SpAs of E17.5 placentae from WT and *Phd2*^–/–^ cKO pregnant mice after treatment with PBS or ACF (scale bars represent 25 μm). (Arrows indicate the location of the walls [W], and the dotted lines delineate inner and outer wall of the maternal SpAs.) L, lumen. (**E**) Wall thickness and lumen area measurements of decidual SpAs in GD17.5 placentae from WT and *Phd2*^–/–^ cKO pregnant mice treated with either PBS or ACF (***P < 0.001 relative to the WT measurements, 1-way ANOVA, Newman-Keuls post hoc test; *n* = 17 SpAs of 6 WT placentae, *n* = 41 SpAs of 12 *Phd2*^–/–^ cKO placentae, *n* = 25 SpAs of 12 placentae from ACF-treated *Phd2*^–/–^ cKO dams). (**F**) Mean arterial blood pressure across gestation in WT and *Phd2*^–/–^ cKO pregnant mice treated with either PBS or ACF (*P < 0.05 relative to the *Phd2^–/–^* cKO pregnant mice at the corresponding gestational age, 1-way ANOVA, Newman-Keuls post hoc test; *n* = 4 separate pregnant dams per condition).

Since our *Phd2^–/–^* cKO model recapitulated many features of E-PE, including increased placental HIF1A protein and activity, we tested its use as a preclinical model for screening therapeutic HIF1 inhibitors. We interrogated the FDA-approved HIF inhibitor ACF for its potential to reverse the preeclamptic phenotype produced by the deletion of *Phd2* in the JZ layer of the placenta (26, 52). We first ascertained the toxicity and/or teratogenicity of ACF in WT (*Phd2^fl/fl^*) pregnant mice. Pregnant dams were subjected to daily intraperitoneal (150 μL) injections of ACF at a dose of 2 mg/kg of body weight from E7.5 to E14.5 (Figure 7A). This dosage of ACF did not induce any fetal loss, and fetal over placental weight ratios at E17.5 were similar in ACF- and PBS-treated mothers (Supplemental Figure 4B). After delivery, postnatal growth of pups from ACF-treated mothers was the same as those of pups from PBS-treated mothers (Supplemental Figure 4C). Gross histopathological examination of E17.5 fetuses by a mouse pathologist blinded to the study did not reveal any noticeable differences between of E17.5 fetuses from mothers treated with PBS or ACF (Supplemental Figure 4D). Additionally, no obvious gross morphological differences were noted between E17.5 placentae from WT pregnant mice and WT pregnant mice treated with ACF (Figure 7A vs. Supplemental Figure 5A). These findings indicate that a daily dose of 2 mg/kg ACF has no detrimental effect on placental, fetal, and postnatal development in healthy pregnant mice.

Next, we subjected *Phd2^–/–^* cKO pregnant mice to the same daily injection regimen of 2 mg/kg ACF (Figure 7A). Control groups included *Phd2^–/–^* cKO and *WT* (*Phd2^fl/fl^*) pregnant mice injected with PBS. ACF treatment corrected the intrauterine fetal loss seen in *Phd2^–/–^* cKO pregnant mice (Supplemental Table 1). Real-time PCR showed that *Vegfa* and *Hyou1* mRNA expression was restored to levels of WT control in *Phd2^–/–^* placentae from pregnant mice treated with ACF (Supplemental Figure 5B), confirming inhibition of HIF1 downstream signaling by ACF. Fetal and placental gross morphology at E17.5 was similar in WT and ACF-treated *Phd2^–/–^* cKO pregnant mice (Figure 7B and Supplemental Figure 5, A and C). H&E staining of E17.5 *Phd2^–/–^* cKO placentae demonstrated a reduced labyrinth and enlarged JZ layer (Figure 7, B and C) like that seen in E14.5 *Phd2*^–/–^ cKO placentae (Figure 2, A and B). Administration of ACF corrected this altered layer distribution in the E17.5 *Phd2*^–/–^ cKO placentae (Figure 7, B and C). Furthermore, ACF attenuated the dilation of maternal sinusoid spaces of the *Phd2^–/–^* cKO placentae (Supplemental Figure 5D). Histological analysis of decidual SpAs showed that ACF restored SpA remodeling of the *Phd2^–/–^* cKO placental bed to that found in the WT placental bed (Figure 7, D and E). Together with these placental improvements, ACF attenuated the overall fetal weight decrease of E17.5 *Phd2^–/–^* cKO embryos (Supplemental Figure 4B). We then investigated whether ACF also improved maternal preeclamptic features. *Phd2^–/–^* cKO pregnant mice treated with ACF had, indeed, lower mean arterial blood pressures across gestation than PBS-treated *Phd2^–/–^* cKO mice (Figure 7F). To determine a dose-response relationship, we also treated *Phd2^–/–^* cKO pregnant mice with either 1 or 4 mg/kg of ACF. Daily administration of 1 mg/kg ACF did not lower the elevated blood pressure of *Phd2^–/–^* cKO pregnant mice (Supplemental Figure 5E). In contrast, daily treatment with 4 mg/kg of ACF reduced the blood pressure to levels of pregnant WT mice injected with PBS (Supplemental Figure 5E). However, inspection of uterine horns at E17.5 of both WT and *Phd2^–/–^* cKO pregnant mice treated with 4 mg/kg of ACF revealed fewer embryos and embryo resorption, suggesting that 4 mg/kg ACF causes developmental toxicity. Since 2 mg/kg was not teratogenic, we analyzed the maternal organs (kidneys, heart) of pregnant mothers treated with that dose. Urine analysis showed that ACF treatment of *Phd2^–/–^* cKO pregnant mice restored the creatinine, albumin, and albumin over creatine ratio as well as protein levels to that of WT pregnant mice (Figure 5, C and D, and Supplemental Figure 1C). H&E staining and TEM of maternal kidneys at 17.5 days of pregnancy showed that glomeruli of ACF-treated *Phd2^–/–^* cKO pregnant mice had similar histological and ultrastructural morphology as glomeruli of WT pregnant mice injected with PBS (Supplemental Figure 3A and Supplemental Figure 4A). Glomerulus area and diameter as well as Bowman space were all restored to those of WT pregnant dams (Supplemental Figure 3B). ACF partially reverted the reduced urinary space and almost completely reverted the endocapillary space in kidney glomeruli of *Phd2^–/–^* cKO pregnant mice back to WT values (Supplemental Figure 4A). In addition, CD31 staining and TEM images confirmed that glomerulus from *Phd2^–/–^* cKO pregnant mothers injected with ACF presented no endothelial cell swelling or increased number compared to the kidneys from WT pregnant mothers (Supplemental Figure 3A). Finally, ACF treatment ameliorated the changes in interfibrillar mitochondrial organization and dynamics in maternal cardiomyocytes of *Phd2^–/–^* cKO pregnant dams (Figure 6, B and C).

**Figure 8:**
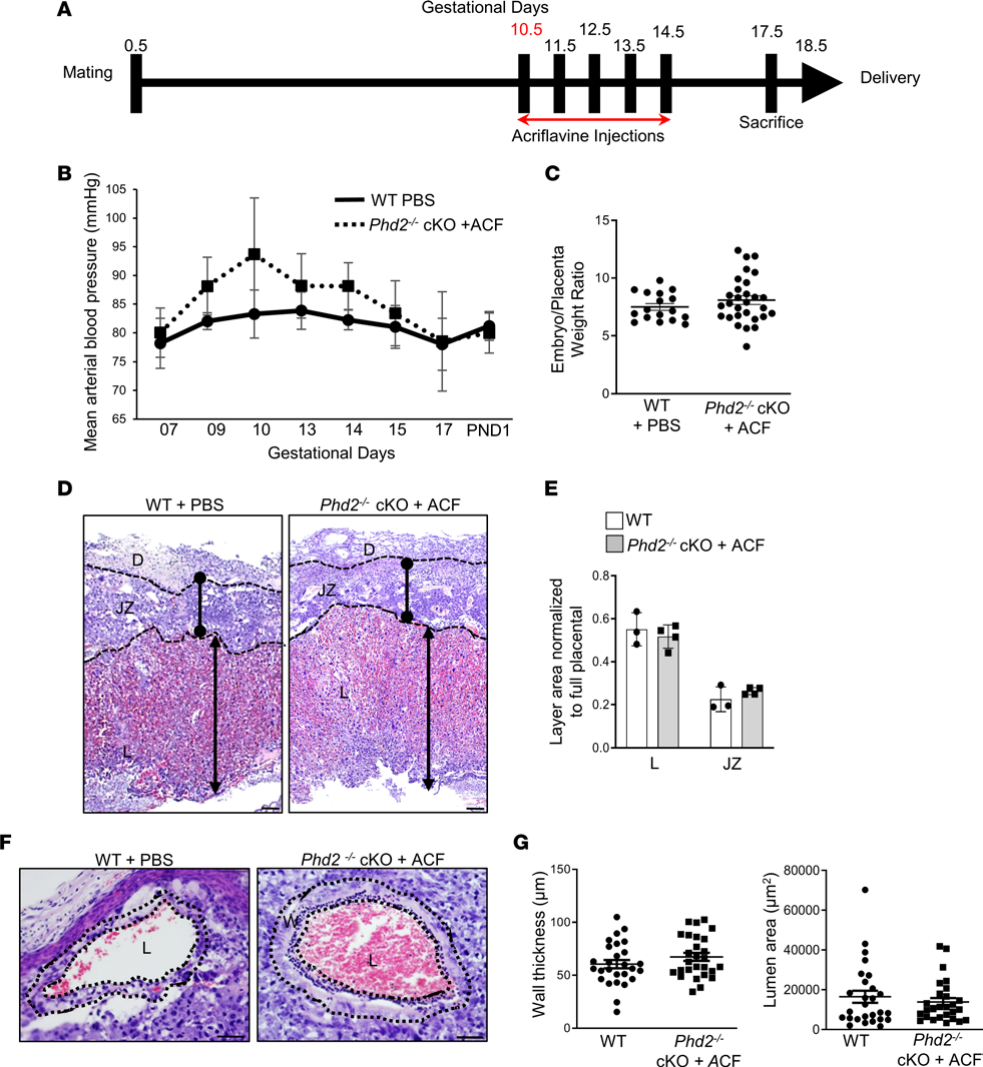
ACF administration at midgestation of pregnancy corrects placental, fetal, and maternal preeclamptic phenotype in JZ-specific *Phd2^–/–^* cKO pregnant mice. (**A**) Schematic of ACF injection regimen during midgestation (GD10.5–14.5) of pregnancy. (**B**) Mean arterial blood pressures across gestation in WT and *Phd2*^–/–^ cKO pregnant mice treated with either PBS or ACF from days 10.5 to 14.5 of gestation (no significant differences relative to WT, 1-way ANOVA, Newman-Keuls post hoc test; *n* = 4 separate pregnant mothers per condition). (**C**) Fetal over placental weight ratios at gestational day 17.5 of ACF- and PBS-treated (GD10.5–14.5) mothers (*n* = 17 embryos of WT pregnant mothers treated with PBS; *n* = 29 embryos of *Phd2*^–/–^ cKO pregnant mothers treated with ACF). (**D**) Representative H&E staining and (**E**) morphometric analysis of E17.5 placental sections from WT and *Phd2*^–/–^ cKO pregnant mice treated with either PBS or ACF from GD10.5 to 14.5 (scale bars represent 100 μm). Size of labyrinth (L) and junctional zone (JZ) are expressed as a percentage of whole placenta. (*n* = 4 WT and 4 *Phd2*^–/–^ cKO placentae.) D, decidua. (**F**) H&E staining of decidual maternal SpAs of E17.5 placentae from WT and *Phd2*^–/–^ cKO pregnant mice after treatment with PBS or ACF from GD10.5 to 14.5 (scale bars represent 25 μm) (dotted lines delineate inner and outer wall of the maternal SpAs). W, wall; L, lumen. (**G**) Wall thickness and lumen area measurements of decidual SpAs in E17.5 placentae from WT and *Phd2*^–/–^ cKO pregnant mice treated with either PBS or ACF from GD10.5 to 14.5 (*n* = 27 SpAs of 6 WT placentae of PBS-treated pregnant mice and *n* = 27 SpAs of 6 *Phd2*^–/–^ cKO placentae of ACF-treated pregnant dams).

Together, these findings indicate that daily administration of 2 mg/kg ACF between E7.5 and E14.5 prevented various placental, fetal, and maternal phenotypic features of E-PE in the JZ-specific *Phd2^–/–^* knockout mice. However, ACF administration was commenced at E7.5 before the adverse phenotype (i.e., elevated maternal hypertension) developed. Therefore, in a second set of experiments, ACF was administered at E10.5 after pathology (i.e., elevated maternal hypertension) was established (Figure 8A). ACF treatment at midgestation of pregnancy (E10.5–E14.5) lowered the elevated mean arterial blood pressures at E10.5 across later gestation to those of PBS-treated WT pregnant mothers (Figure 8B). Also, it corrected fetal growth (Figure 8C vs. Supplemental Figure 4C) and placental dysmorphology (Figure 8, D and E, vs. Figure 2B) and restored SpA remodeling of the *Phd2^–/–^* cKO placental bed (Figure 8, F and G, vs. Figure 3B). These data suggest that ACF treatment at midgestation of pregnancy can rescue and correct established preeclampsia in JZ-specific *Phd2^–/–^* cKO pregnant mice.

## Discussion

In the present study, we demonstrate that deletion of *Phd2* in the JZ of the placenta of pregnant mice provokes a pregnancy phenotype that resembles early-onset severe preeclampsia in humans. Conditional *Phd2* removal during pregnancy reproduces chronic uteroplacental hypoxia, hallmarked by elevated placental levels of HIF1A (10). Ensuing aberrant HIF1-mediated transcriptional activities result in dysmorphic placentation (expansion of JZ, compaction of labyrinth, dilation of maternal sinusoids) and impaired maternal SpA remodeling that culminates in fetal growth restriction. Moreover, pregnant dams develop hypertension, renal and heart pathology, as well as proteinuria, all classic hallmarks of early-onset severe preeclampsia in humans (2, 20–22, 24). Of clinical relevance, we report prevention and rescue of placental, fetal, and maternal features of E-PE in the *Phd2*^–/–^ cKO using a small molecule HIF1 inhibitor.

Mice and humans undergo hemochorial placentation with intrauterine trophoblast cell invasion and trophoblast**-**directed SpA remodeling. Despite structural differences, transgenic mouse models have progressed the development of preclinical models of preeclampsia (6, 7), albeit none of them encompass all pathophysiological changes associated with severe preeclampsia. KO mice of endothelial nitric oxide synthase (*Nos3*) are hypertensive prior and, during pregnancy, have proteinuria, uterine artery dysfunction, and fetal growth restriction, but they lack placental abnormalities (53). Mice overexpressing renin-angiotensin system components (54, 55) are hypertensive before pregnancy, making them, like *Nos3-*deficient mice, a more suitable model for women who enter pregnancy hypertensive. Transgenic mice overexpressing the human storkhead box 1 (*STOX1*) gene in the placenta exhibit many preeclamptic features, including increased blood pressure (35, 56), but a limitation of this model is that hypertension develops at day 3 of pregnancy, which is much earlier than reported in preeclamptic women and prior to establishment of the mature mouse placenta around E10.5 (35). Overexpression of soluble fms-like tyrosine kinase (sFlt1) in the placenta of pregnant mice causes late-onset hypertension, reduced pup weights, and proteinuria, but placental defects besides reduced vascularization of the labyrinth are limited (57). Likewise, BPH/5 mice exhibit key pathophysiological features seen in human preeclampsia (58); however, BPH/5 mice are hypertensive prior to pregnancy, and all preeclamptic features develop late in pregnancy, suggesting the mice are more suitable as a model of superimposed or late preeclampsia (58). In human preeclampsia, HIF-1 is increased due to placental hypoxia, a key feature of preeclampsia (19). Various murine models have interrogated the role of hypoxia in development of preeclampsia, including mice overexpressing HIF1A (21) and mice deficient in catechol-O-methyltransferase (20) that produce the HIF1A inhibitor 2-methoxyestradiol. Both models recapitulate multiple aspects of preeclampsia, namely incomplete remodeling of maternal SpAs, fetal growth restriction, hypertension, and proteinuria. However, global overexpression or deletion of gene of interest confound the maternal measurements in both models. Producing a placenta-specific gene deletion is challenging. No reliable cre-transgenic deleter mouse for ubiquitous deletion of *LoxP*-flanked sequences in all layers of the mouse placenta is currently available. The GCM1-cre mouse allows for gene deletion in the labyrinthine layer (59) while the Tpbpa-cre deleter targets the JZ for removal of floxed sequences (38). In the present study, we selected the Tpbpa-cre (4311-cre) deleter because we found prominent PHD2 expression in the JZ of the mouse placenta. Tpbpa is expressed during early placental development in the ectoplacental cone and later on in SpT cells of the JZ that are regarded as progenitors of glycogen cells and TGCs (38, 60). Our JZ-specific *Phd2^–/–^* cKO knockout exhibited similar placental changes within the labyrinth as seen in global *Phd2*^–/–^ cKO pregnant mice (18). The size of the labyrinth was reduced, its vasculature was disrupted, and abnormal invasion of SpTs and mislocalization of glycogen cells into the labyrinth occurred. Moreover, the conditional *Phd2* deletion reduced SpA-TGC invasion and SpA remodeling, culminating in early-onset fetal growth restriction. Fetuses of global *Phd2^–/–^* cKO pregnant mice succumbed at E12.5–E14.5 (18). In contrast, *Phd2^–/–^* cKO mice displayed no embryonic lethality, thereby allowing for fetal and maternal assessment during pregnancy. The *Phd2^–/–^* cKO pregnant mice developed early new-onset hypertension during pregnancy as well as renal and myocardial pathology and proteinuria. Maternal hypertension subsided after birth. Similar preeclampsia-like features were observed in mice overexpressing constitutively active HIF1A in trophoblasts, except for early-onset fetal growth restriction (24). Thus, our murine model of placental excess of HIF1A due to removal of *Phd2* in the JZ not only replicates the reduction of placental PHD2 in human E-PE (3) but also reproduces most of its key clinical features (1), underscoring its use as a preclinical model of E-PE. Our preeclamptic *Phd2*^–/–^ cKO mouse model showed sustained HIF1A expression in the placenta. Besides hypoxia, HIF1A is triggered by ROS, cytokines, and a variety of metabolic stimuli and signaling pathways (61). Hence, it would be of interest to examine whether upstream regulatory mechanisms of HIF1A are triggered in our mouse model.

In the present study, we determined blood pressure using a tail-cuff method. There is an ongoing debate between telemetry versus tail-cuff measurements. Telemetry significantly increases mouse mortality and morbidity (62) and requires accurate surgery from skilled staff. Tail-cuff measurements may cause animal stress (62, 63) and may underestimate blood pressure changes that are due to handling and restraints. However, comparison of tail-cuff and telemetry on rested mice showed that both methods produce similar results (64). To ensure fair comparison, we trained our female mice daily on a heated restraining platform for a week prior to mating, and all measurements (15 to 25 measurements per session) were done each time simultaneously on paired WT and *Phd2^–/–^* cKO pregnant mice. Therefore, any stress induced from handling and measurement is similar for our paired experimental groups.

Besides preventive therapies with daily low-dose aspirin and/or calcium, there are no effective treatments of preeclampsia except for preterm delivery. Potential therapeutic interventions with alpha-1 microglobulin and pravastatin have been reported in *STOX1* (65), *C1q* (66), and *sFlt1* (57) preeclamptic mouse models, respectively. Although excess HIF1A is a key feature of E-PE (3, 10, 11), pharmacological inhibition of the HIF pathway during pregnancy has been limited. Knockdown of *Hif1a* mRNA with specific siRNA in pregnant mice infused with angiotensin II type I receptor agonistic autoantibodies (AT_1_-AAs) attenuated AT_1_-AA–induced PE features (23). However, the occurrence of AT_1_-AA is not restricted to pregnancy (67), and injection of AT_1_-AA into nonpregnant mice also results in hypertension (68). In addition, HIF upregulation by AT_1_-AA infusion is not restricted to the placenta, which confounds the RNA interference findings. The HIF pathway has multiple target points for therapeutic intervention (e.g., HIF synthesis, degradation, and activation), but most HIF inhibitors tested so far lack specificity (25). Recently, a new group of more selective HIF inhibitors that target the dimerization of HIF subunits has been identified (69). They interfere with the dimerization process and reduce HRE binding. The acridine derivative ACF was identified in a drug screen of 200 compounds for their potential to inhibit HIF heterodimerization (26). Originally, ACF was used to combat parasites and bacterial infections (70). ACF is an FDA-approved drug for human topical (non-oncological) uses in wound healing that is repurposed because of its antitumor properties. Ample studies have reported ACF being a safe and promising HIF1-targeting therapy for a variety of solid tumors (52, 71–74). Moreover, ACF has been identified as a potential treatment for ocular neovascularization (75). Based on these favorable reports, we investigated its potential to counteract HIF1-induced placental dysmorphologies that lead to E-PE in our *Phd2^–/–^* cKO mice. ACF’s nonselective inhibition of both HIF1 and HIF2 (26) was no concern as we only found HIF1 being upregulated in the placentae of *Phd2^–/–^* cKO mice. Interestingly, we did not observe changes in blood pressure in WT pregnant animals injected with ACF, suggesting that this drug does not affect vascular beds outside of the placenta. We observed that daily administration of 2 mg/kg ACF at E7.5–E14.5 prevented most placental, fetal, and maternal preeclampsia-like features seen in the pregnant *Phd2^–/–^* cKO mice. It corrected placental abnormalities including SpA remodeling and associated fetal growth restriction. Also, it reduced maternal hypertension and attenuated maternal renal and cardiac pathology. Importantly, ACF treatment of WT pregnant mice had no detrimental effects on placental and fetal development and did not affect maternal health. Of clinical relevance, similar outcomes were obtained when ACF treatment was started at midgestation of pregnancy (E10.5–E14.5; mimics early third trimester treatment in humans) after maternal hypertension was established. We noted that ACF injections either from E7.5–14.5 or from E10.5–E14.5 were not able to fully restore the blood pressure to its WT levels, suggesting that dosing and time of intervention need further fine-tuning. Ideally, HIF1 inhibitors should be administered when clinical symptoms of early preeclampsia manifest. This would not only ameliorate maternal symptoms but could also delay early delivery of the baby and prevent associated comorbidities.

A limitation of our preclinical mouse model is that preeclampsia is induced via genetic ablation of a single gene in the placenta, in contrast to humans where preeclampsia is a multifactorial disease that occurs spontaneously. Although our experimental *Phd2^–/–^* cKO model exhibits many clinical features of preeclampsia, it may not reflect all aspects of human preeclampsia. Evaluating ACF in other HIF1-induced PE models such as the reduced uterine perfusion pressure (RUPP) rat model (76) that is closer to humans when investigating trophoblast cell invasion into SpAs (77) could bring additional valuable information about the mode of action of ACF. However, the RUPP model is more suitable for L-PE as the surgical intervention to restrict blood flow is generally conducted during the last week of gestation. African green monkeys have been reported to spontaneously develop gestational hypertension with proteinuria and fetal growth restriction during pregnancy (78). Once this monotocous preclinical preeclampsia model is further characterized, including placental upregulation of HIF1, it could be used for evaluating HIF1 inhibitors in a spontaneous model of preeclampsia. Another limitation is that we only employed ACF as a small molecule HIF1 inhibitor and that it may have off-target effects. The cyclic peptide cyclo-CLLFVY is another inhibitor of HIF1 heterodimerization (79). While it is not FDA approved, it would be worthwhile to confirm the therapeutic benefits of targeting HIF1 in the *Phd2^–/–^* cKO model of preeclampsia with this peptide. ACF has been reported to inhibit argonaute 2 (Ago2), an endonuclease involved in the microRNA processing through the RNA-induced silencing complex (80). Ago2 inhibition with miRNA-15b has been reported to reduce trophoblast cell invasion and endothelial cell tube formation in vitro (81). However, our observation that daily ACF treatment had no detrimental effect on placental, fetal, and postnatal development in healthy pregnant mice argues against a major role of Ago2 in placentation. In cancer cells, ACF has been shown to inhibit the unfolded protein response (UPR) pathway via inhibition of eukaryotic translation initiation factor 2A (eiF2a) phosphorylation and downregulation of the activating transcription factor 4 (ATF4) transcriptional program (82). ATF4 is one of the central transcription factors in the UPR induced by severe hypoxia, independent of HIF signaling (83). In pilot experiments, we found no change in phosphorylated eiF2a, 78 kDa glucose regulated protein (GRP78), and ATF4 expression between placentae of *Phd2^–/–^* cKO embryos and littermate controls (Supplemental Figure 1D and full, uncut gels), suggesting no UPR activation in *Phd2^–/–^* cKO placentae. Thus, it is unlikely that ACF rescued the E-PE phenotype in these pregnant *Phd2^–/–^* cKO mice via inhibition of this pathway. Systemic administration of ACF during development is the third limitation of the current study. HIF regulates many genes in various developing organs, and we have only assessed gross morphology of the fetus after ACF treatment. Targeting the placenta needs to be considered. Recent studies have suggested that tumor-homing peptide-coated nanoparticles (liposomes) loaded with HIF1 inhibitors could be used for targeted delivery to the placenta (84).

In summary, while preeclampsia is a spontaneous and multifactorial disease, placental hypoxia and associated HIF1A expression are key features of severe preeclampsia. In that context, as suggested by our results, targeting the HIF pathway in pregnant women at risk of developing or having E-PE could be of immense value for improving maternal and progeny health.

## Methods

### Study design.

The objective of this study was to produce a mouse model of preeclampsia that would be amenable to preclinical testing of HIF1 inhibitors. Sample size for all experiments performed in this study ranged from *n* = 4–10 primiparous pregnant animals per group (multiple placentae were considered intra-animal replicates). All experiments were repeated at least 3 times, mice of similar age were used for each experimental group (6 to 15 weeks of age), and abnormally large or small litters were excluded from the study. Where applicable, morphometric analyses were conducted by 2 individuals blinded to the study.

### Mice.

*Phd2^fl/fl^* (Stock Egln1tm1Kael/J) mice were obtained from The Jackson Laboratory while *4311^cre-EGFP^* (*Tpbpa^cre-EGFP^*) mice were from the Canadian Mouse Mutant Repository (The Hospital for Sick Children, Toronto, Ontario, Canada). *Phd2^fl/fl^* mice (27) were bred with homozygous *4311^cre-EGFP^* (*Tpbpa^cre-EGFP^*) mice to produce *Tpbpa^cre^ Phd2^fl/fl^* mice. Following this, homozygous *Tpbpa^cre^ Phd2^fl/fl^* males were selected to mate with female *Phd2*^fl/fl^ mice to generate JZ*-*specific *Phd2^–/–^* cKO (*Tpbpa^cre^ Phd2^–/–^*) embryos (28), while control WT embryos were produced by breeding male *Phd2^fl/fl^* mice lacking *Tpbpa^cre^* with female *Phd2^fl/fl^*. No issues with fertility were noted. Pregnant animals were sacrificed at E14.5 and E17.5 using CO_2_ euthanasia. Upon sacrifice, dissection was performed, and maternal kidneys, placentae, and embryos were collected, weighed, and fixed in paraformaldehyde or snap-frozen for genotyping and histological and molecular analyses.

### Acriflavine injections.

Pregnant *Phd2^–/–^* cKO (*Tpbpa^cre^* *Phd2^fl/fl^*) and control WT (*Phd2^fl/fl^*) mice received acriflavine (dissolved in PBS) via daily intraperitoneal injections (150 μL) at a dose of 2 mg/kg body weight. Control WT and *Phd2^–/–^* cKO pregnant mice received an equivalent volume of PBS alone. Intraperitoneal injections occurred once a day at the same time between E7.5 and E14.5 (Figure 6A) or E10.5 and E14.5 (Figure 8A). Sacrifice, dissection, and organ collection were performed as described above.

### Blood pressure measurements.

Prior to mating, female *Phd2^fl/fl^* mice were accustomed to human handling and blood pressure measurement for at least a week. They were then allowed to rest for 2 days without measurements to ensure maximum efficiency of breeding with male *Tpbpa^cre^ Phd2^fl/fl^* mice. Once a vaginal plug was observed, the pregnant dams were set aside, and blood pressure was measured daily at the same time (approximately 2 pm). Blood pressure was monitored using a noninvasive CODA mouse tail-cuff apparatus that measures the volume-pressure in awake animals (85). Briefly, individual pregnant mice were placed in a Plexiglas restrainer for 5 minutes on a body temperature–heated platform with the tail passing through a tail-cuff sensor. Blood pressure was then measured as a series of 15 to 30 inflation-deflation cycles of the tail-cuff, yielding ten 45-second measurements, for a total of 25 minutes (1 experimental set is composed of 5 minutes acclimation, 5 cycles of acclimation measurements, 15–20 cycles of measurements).

### Urine collection and creatinine and protein analysis.

Maternal urine was collected by restraining mice above a Petri dish, which provoked spontaneous urination. Urine creatinine content was measured using a creatinine assay kit (Crystal Chem). Urine albumin was measured using a mouse albumin elisa kit (Abcam Inc.). Urine protein content was estimated using Uristix (Siemens Healthcare Diagnostic Inc.) by adding a drop of undiluted urine and color-reading the sticks 60 seconds later.

### Transmission electron microscopy.

Placentae and maternal kidneys and hearts were fixed in 2% (v/v) glutaraldehyde in 0.1 M cacodylate buffer (pH 7.3) and were further processed for TEM at the Nanoscale Biomedical Imaging Facility at The Hospital for Sick Children, Toronto. Images were captured with a FEI Technai 20 electron microscope. Morphometric analysis of maternal kidney urinary space and capillary lumen was performed using ImageJ (version 1.50i, NIH). Mitochondrial shape and size of maternal heart muscle were also analyzed using ImageJ software by manually tracing only clearly discernable outlines of mitochondria as previously described (50).

### Histology, IF, and immunohistochemistry.

Placentae, fetuses, and maternal kidneys and hearts were fixed in 4% (v/v) paraformaldehyde, dehydrated in an ascending series of ethanol, and transferred to xylene prior to embedding in paraffin and sectioning. Thin 5 μm sections were rehydrated and used for histological, IF, and IHC analyses. For gross histology, sections were stained with H&E, PAS, or MAS. For IF, heat-induced antigen retrieval with 10 mM citrate buffer, pH 6.0, was performed. Sections were then blocked with 5% (v/v) horse serum and incubated with primary antibody diluted in antibody diluent (0.4% v/v sodium azide, 0.625% *w/v* gelatin in PBS) containing 5% (v/v) horse serum at 4°C overnight. Sections were then incubated with secondary antibody (see below), counterstained with DAPI, and mounted on slides. Images were viewed and captured using Leica SD6000 spinning disk confocal microscope. For immunohistochemistry, sections were treated with 3% (v/v) H_2_O_2_ in methanol for 30 minutes at room temperature to block endogenous peroxidase activity. Sections were then incubated with primary antibody overnight at 4°C followed by biotinylated secondary antibody for 2 hours at room temperature. Sections were washed with PBS, then incubated with avidin/biotin-horseradish peroxidase complex (VectaStain ABC Standard Kit; Vector Laboratories), and formed complexes were identified with DAB substrate (0.075% [WT] DAB in PBS containing 0.002% [v/v] H_2_O_2_). Slides were mounted using Surgipath micromount medium (Leica). Images were captured using an Olympus BX61 motorized light microscope system.

### Morphometry analyses.

Morphometric analyses of placentae and maternal kidneys were performed using ImageJ software (version 1.50i). Placental layer distribution was assessed using full placental sections stitched from high-resolution images taken at 10× original magnification. Using ImageJ software scaling and circling tools, total placenta, JZ, and labyrinthine areas were measured. The decidual layer area was calculated from the other measurements (i.e., D area = total area – [L area + JZ area]). SpAs were identified in the decidual layer based on their location and morphology. Very large and small vessels were excluded from the analysis as they could not be measured properly using ImageJ circling tools. The SpA inner and outer perimeters were delineated and their areas were extracted. Diameters were calculated using the D = r^2^ formula, and wall thickness was obtained by subtracting inner and outer diameter values. Kidney glomeruli were identified within the kidney sections, the Bowman capsule and the glomerular perimeters were delineated, and their area and diameter were extracted from the measurements. Bowman space area was calculated by subtracting the Bowman capsule and glomerulus areas. ImageJ software was also applied to glomeruli TEM images to determine endocapillary-free space, which was calculated by subtracting the outer diameter of the capillary vessels and the inner material contained within vessels. All measurements were performed independently by 2 investigators blinded to the samples but not to the study.

### Quantitative PCR.

Real-time PCR was performed as previously described (3). Total RNA was extracted from placental tissues using RNeasy Plus Mini Kit (QIAGEN). One μg of total RNA was reverse-transcribed using qScript cDNA Synthesis Kit (Quantabio). The resulting cDNA was quantified by real-time PCR (CFX96 Real-Time System, Bio-Rad) using PerfeCTa FastMix II from Quantabio and mouse-specific TaqMan (Assays-on-Demand) probes targeting *18S* (Mm03928990_g1), *Vegfa* (Mm01281449_m1), and *Hyou1* (Mm00491279_m1), purchased from Applied Biosystems (Thermo Fisher Scientific). For each probe, a dilution series determined the efficiency of amplification of each primer set. Gene expression was normalized to *18S*, expressed as the relative fold change using the ΔΔCt method, and compared with selected appropriate positive or negative controls.

### Lactate analysis.

Placental samples were analyzed at the Analytical Facility for Bioactive Molecules of The Hospital for Sick Children, Toronto, Ontario, Canada. Placentae were homogenized in 15% ice-cold 10 mM phosphate buffer plus 85% ethanol using a Precyllys Tissue homogenizer. For quantitative analysis a separate standard curve of lactate was generated. Samples and standards were filtered, derivatized, and subjected to liquid chromatography-tandem mass spectrometry using an Agilent 1290 HPLC coupled to a SCIEX 5500 mass spectrometer. Data were acquired and analyzed using Analyst v.1.6.3 (SCIEX).

### Antibodies.

Mouse anti-mouse monoclonal PLF (catalog sc-271891) (IF [1:1,000]), mouse anti-human monoclonal CD34 (sc-7324), mouse anti-human monoclonal GRP78 (sc-376768) (Western blot [WB] [1:500]), and mouse anti-chicken monoclonal ACTB (sc-47778) (WB [1:2,000]) antibodies were purchased from Santa Cruz Biotechnology. Mouse anti-mouse monoclonal HIF1A (catalog NB100-105) (WB [1:50]) and rabbit anti-mouse polyclonal PHD2 (NB100-137) (WB [1:1,000]) and HIF2A (NB100-122) (WB [1:1,000]) antibodies were purchased from Novus Biologicals (Bio-Techne). Rabbit anti-human monoclonal AMOT (D204H) (IF [1:100]), rabbit anti-mouse monoclonal CD31 (Pecam, 77699s) (IHC [1:250]), rabbit anti-mouse monoclonal ATF4 (11815) (WB [1:1,000]), and rabbit anti-human monoclonal phosphorylated eIF2a (catalog 3597) (WB [1:1,000]) were purchased from Cell Signaling Technology while rabbit anti-mouse polyclonal HIF2A (catalog ab199) (WB [1:1,000]) and rabbit anti-mouse polyclonal TPBPA (catalog ab104401) (WB [1:1,000]; IHC [1:200]) were from Abcam. DBA (catalog B-1035-5) (IHC [1:500]) was purchased from Vector Laboratories. Secondary horseradish peroxidase–conjugated donkey anti-mouse and goat anti-rabbit IgG (WB [1:2,000]) antibodies were obtained from The Jackson Laboratory (catalog 111-035-146 and 111-035-144, respectively). Secondary Alexa Fluor 488 donkey anti-mouse (catalog A21202) and donkey anti-rabbit IgG (catalog A21206) and Alexa Fluor 594 donkey anti-mouse IgG (catalog A21203) (IF 1:200) antibodies were purchased from Invitrogen. Horse anti-mouse (BA-2000) (IHC [1:200]) and horse anti-rabbit (BA-1100) (IHC [1:200]) biotinylated secondary antibodies were purchased from Vector Laboratories.

### Western blotting.

WB analyses were performed as previously described. Briefly, protein lysates (ranging from 25 to 200 μg) were diluted in sample buffer (10% v/v glycerol, 2% v/v SDS, 5% v/v β-mercaptoethanol, 0.0025 bromophenol blue, 0.06 M Tris base) to a final concentration of 1 μg/μL. A total of 50 μg of protein lysate was then subjected to SDS-PAGE, transferred to PVDF membranes, and immunoblotted as previously described. Protein quantification was performed using ImageJ software, and intensity of the band of interest was normalized to the corresponding β-actin (ACTB) signal.

### Statistics.

Statistical analyses were performed using GraphPad Prism 5 software, and significance was established using a nonparametric unpaired 2-tailed Student’s *t* test or 1-way ANOVA with post hoc Dunnett’s or Newman-Keuls test where applicable. Statistical outliers were identified by performing Grubbs’ test using GraphPad Prism 5. Statistical significance was defined as *P* < 0.05. All data are represented as mean ± SEM of 3 to 6 separate pregnant mice condition.

### Study approval.

All procedures involving animals were performed in compliance with the Animals for Research Act of Ontario and the Guidelines of the Canadian Council on Animal Care. The Centre for Phenogenomics (TCP) Animal Care Committee (Toronto, Ontario, Canada) reviewed and approved all procedures conducted on animals at TCP (AUP 19-0286).

## Author contributions

IC and MP conceived and designed the research study. JS, CP, SA, TP, RL, MK, and AF conducted experiments and acquired data. JS, SA, CP, MP, and IC analyzed data. JS, SA, CP, MP, and IC wrote and reviewed the manuscript.

## Supplementary Material

Supplemental data

## Version 1 10/13/2022

In-Press Preview

## Version 2 12/08/2022

Electronic publication
