# Supplementary material for: HIF1 inhibitor acriflavine rescues early-onset preeclampsia phenotype in mice lacking placental prolyl hydroxylase domain protein 2
Source: JCI Insight. 2022 Dec 8;7(23):e158908. doi: 10.1172/jci.insight.158908 (PMC9746916; doi:10.1172/jci.insight.158908)

	WT (n=12)	*Phd2*$^{-/-}$cKO (n=14)	*Phd2*$^{-/-}$cKO + ACF (n=5; GD7.5-14.5)
Sex	M: 50.7 %<br>F: 49.3 %	M: 48.5 %<br>F: 51.5 %	M: 48.7 %<br>F: 51.3 %
Litter size	9.5± 0.3	7.7 ± 0.5*	8.8 ± 0.2

Table S1: Fetal sex and litter size of WT, *Phd2*$^{-/-}$ and *Phd2*$^{-/-}$ pregnant mice treated with ACF. ACF treatment: 2mg/kg; *p< 0.05 relative to the WT pregnant mice, one-way ANOVA, Neuman-Keuls posthoc-test. M: Male; F: female.

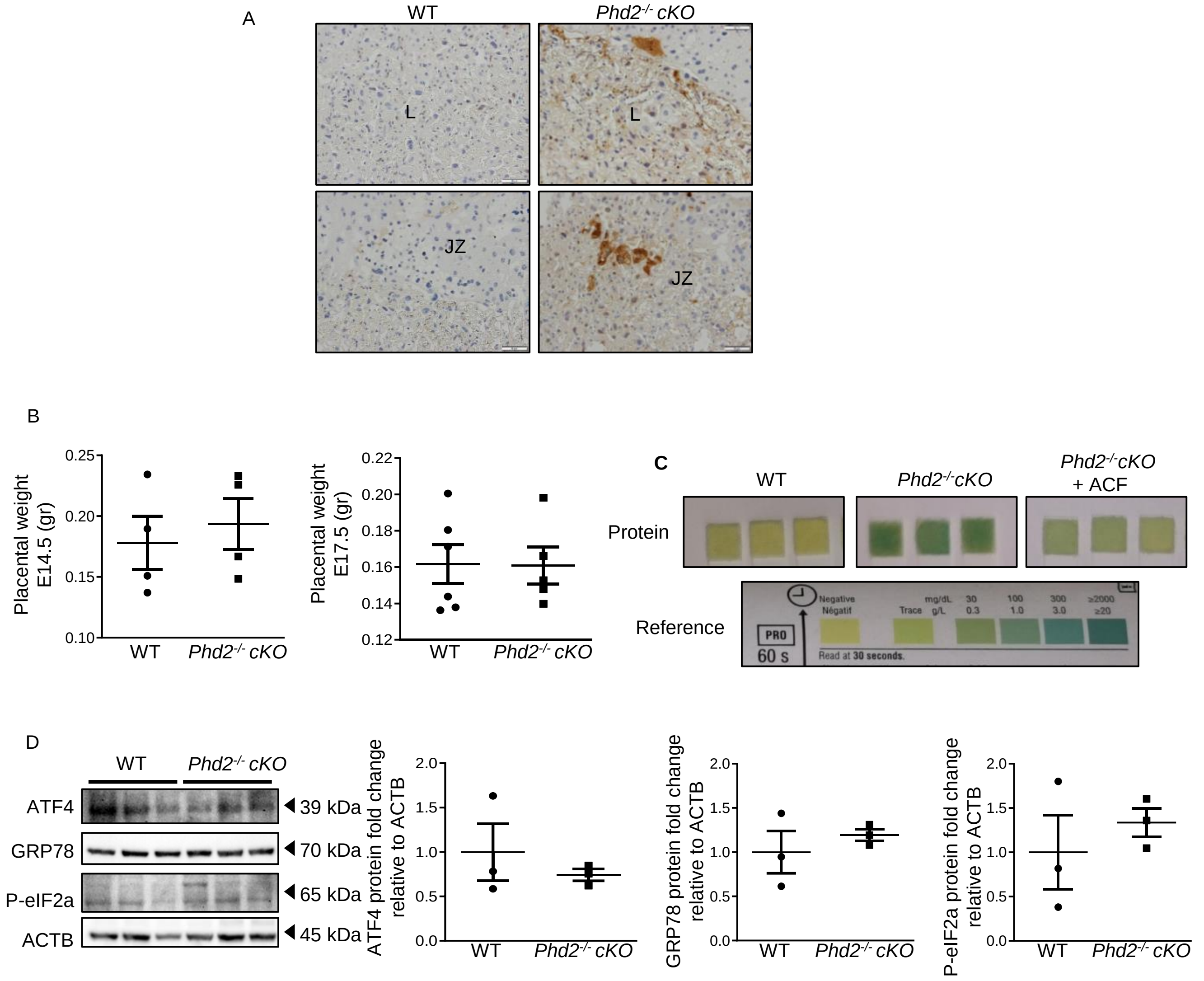

Fig. S1. (**A**) Representative immunohistochemical staining for Hypoxyprobe™ indicating areas of hypoxia (positive brown staining for piminidazole adducts) within the placental labyrinth and junctional zone layers in *Phd2*$^{-/-}$ cKO mice (Scale bars represent 50µm). **(B)** WT and *Phd2*$^{-/-}$ placental weights at gestational day14.5 (n=4 WT litters, 38 placentae and n=4 *Phd2*$^{-/-}$cKO litters, 37 placentae) and 17.5 (n=7 WT litters, 71 placentae and n=5 *Phd2*$^{-/-}$cKO litters, 52 placentae). **(C)** Representative Uristix® measurements of protein in urine collected at day 17.5 of pregnancy from WT and *Phd2*$^{-/-}$ cKO pregnant mice and *Phd2*$^{-/-}$ cKO pregnant mice treated with ACF during early (GD7.5-14.5) pregnancy (urine of 3 separate pregnant mice per condition**).** (**D**) Representative immunoblots for ATF4, GRP78, P-eIF2a and associated densitometry in whole placental lysates from WT and *Phd2*$^{-/-}$ cKO placentae (n=3 WT and n=3 *Phd2*$^{-/-}$cKO placentae).

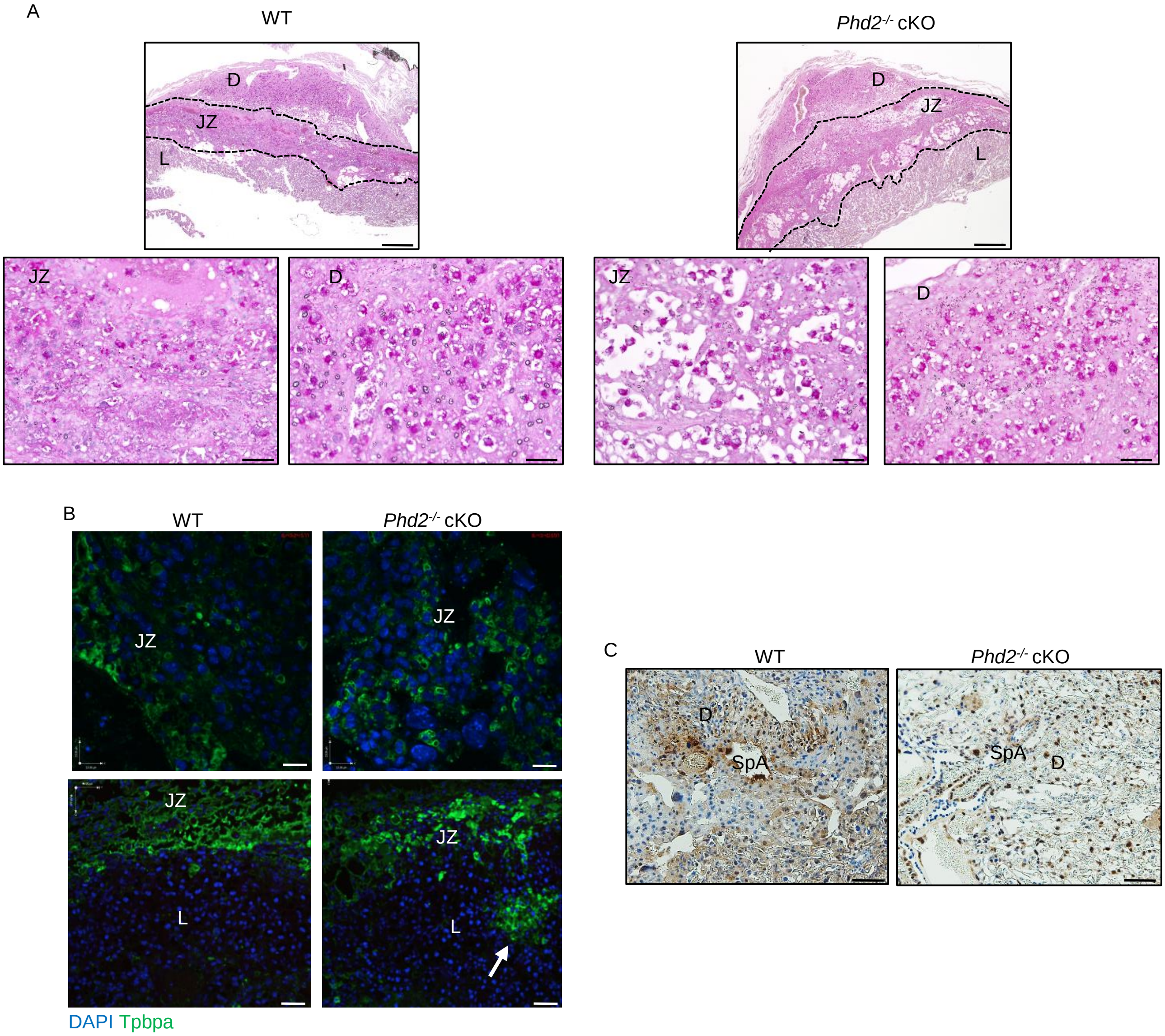

Fig. S2. (**A**) Representative PAS staining of WT and *Phd2*$^{-/-}$ cKO placentae at gestational day 14.5 (Scale bars represent 500µm). Higher magnifications (Scale bars represent 50µm) to visualize PAS-positive glycogen cells in JZ and decidua. D: decidua; JZ: junctional zone; L: labyrinth. **(B)** Representative immunofluorescence staining for *Tpbpa*-positive sphongiotrophoblast (SpT) in JZ (top panels) and labyrinth (bottom panels) of GD14.5 WT and *Phd2*$^{-/-}$ cKO placentae; nuclei were visualized with DAPI (scale bars represent 25 µm). JZ: Junctional Zone. L: labyrinth. Arrow: SpT islets in labyrinth. sphongiotrophoblast (SpT) in JZ (top panels) and labyrinth (bottom panels) of GD14.5 WT and *Phd2*$^{-/-}$ cKO placentae. (**C**) Representative immunohistochemistry for CD69 positive uNKcells in sections from WT and *Phd2*$^{-/-}$ cKO decidua (Scale bars represent 50µm).

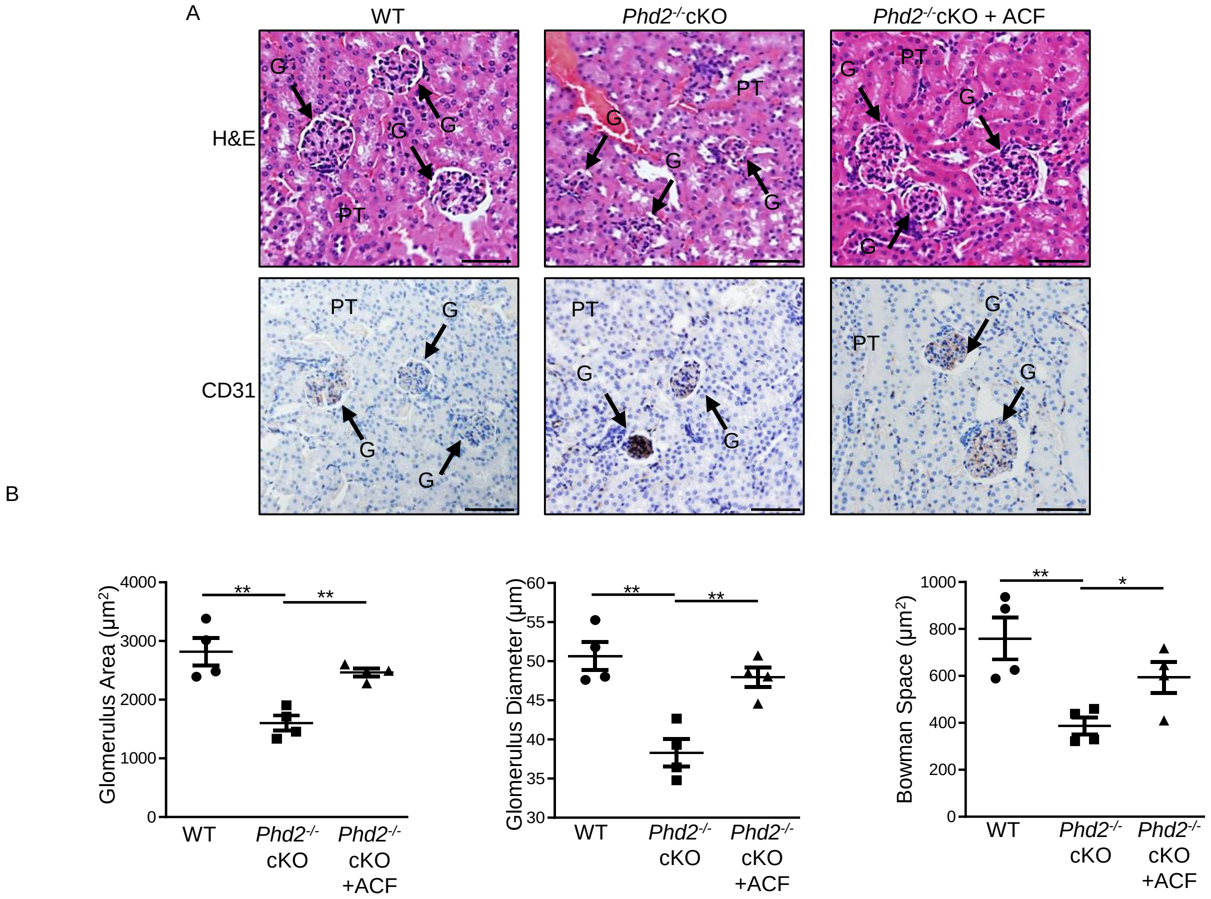

Fig. S3. (**A**) Representative H&E-stained (top panel) and CD31-immunostained (bottom panel) maternal kidneys at day 17.5 of pregnancy of WT and *Phd2*$^{-/-}$ cKO pregnant mice treated with either PBS or ACF. G: glomeruli; PT: proximal tubules. (scale bars represent 50µm). (**B**) Morphometry analyses of glomerulus area and diameter, and Bowman space area in E17.5 kidneys from WT and *Phd2*$^{-/-}$ cKO pregnant mice treated with either PBS or ACF (**p<0.01 relative to the WT measurements, one-way ANOVA, Neuman-Keuls posthoc-test, n=32 glomeruli of 4 kidneys from WT pregnant dams, n=53 glomeruli of 4 kidneys from PBS treated *Phd2*$^{-/-}$ cKO pregnant dams, n = 48 glomeruli of 4 kidneys from ACF treated *Phd2*$^{-/-}$ cKO pregnant dams).

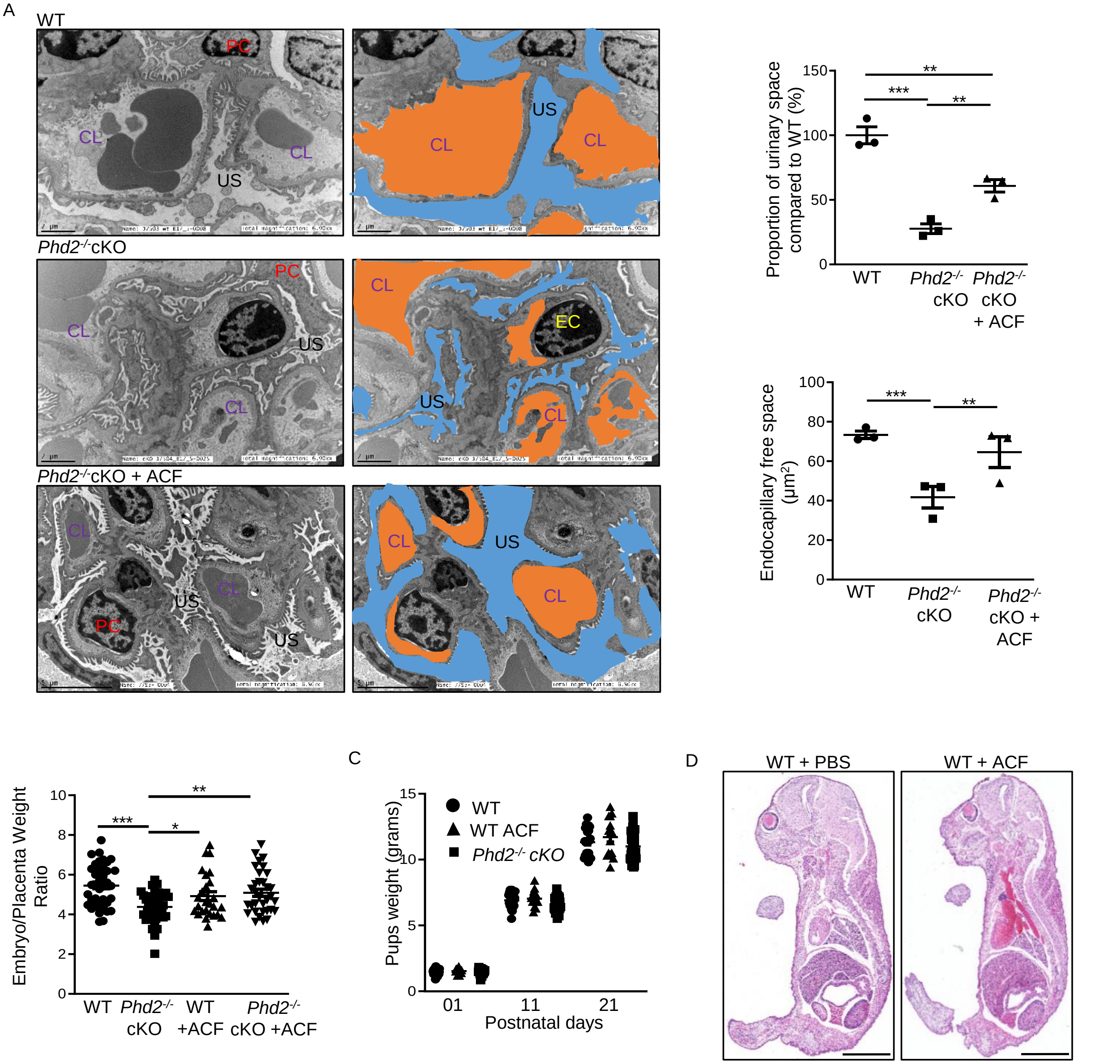

Fig. S4. (A-left panels) Representative TEM images of glomeruli from maternal kidneys at day 17.5 of pregnancy of WT and *Phd2*$^{-/-}$cKO pregnant mice treated with either PBS or ACF. US: urinary spaces; CL: capillary loop; PC: podocytes; EC: endothelial cells. (**A-right panels**) Morphometric analyses of the urinary space and open endocapillary space in maternal kidney glomeruli at gestational day 17.5 of pregnancy of WT and *Phd2*$^{-/-}$ pregnant mice treated with either PBS or ACF. Urinary space is expressed as a percentage of total glomeruli area (*p<0.05 relative to WT, one-way ANOVA, Neuman-Keuls posthoc-test, n=18 glomeruli of 3 separate WT pregnant mice, n=23 glomeruli of 3 separate *Phd2*$^{-/-}$cKO pregnant mice, n=16 glomeruli of 3 separate *Phd2*$^{-/-}$cKO pregnant mice treated with ACF). (**B**) Fetal over placental weight ratios at E17.5 of ACF- and PBS-treated (GD7.5-14.5) mothers (*p< 0.05, **p< 0.01, ***p< 0.001, one-way ANOVA, Neuman-Keuls posthoc-test, n=38 WT embryos, n=46 *Phd2*$^{-/-}$cKO embryos, n=26 embryos of ACF-treated WT pregnant dams, n=32 embryos of ACF-treated *Phd2*$^{-/-}$cKO pregnant mothers); **(C)** Postnatal growth of pups (weights at PND1, 11, 21) from WT mothers injected during early pregnancy (from GD7.5 to 14.5) with either PBS or 2 mg/kg ACF. (**D**) Representative mid-sagittal H&E-stained sections of E17.5 embryos from WT mothers treated with either PBS or 2 mg/kg ACF (scale bars represent 25mm).

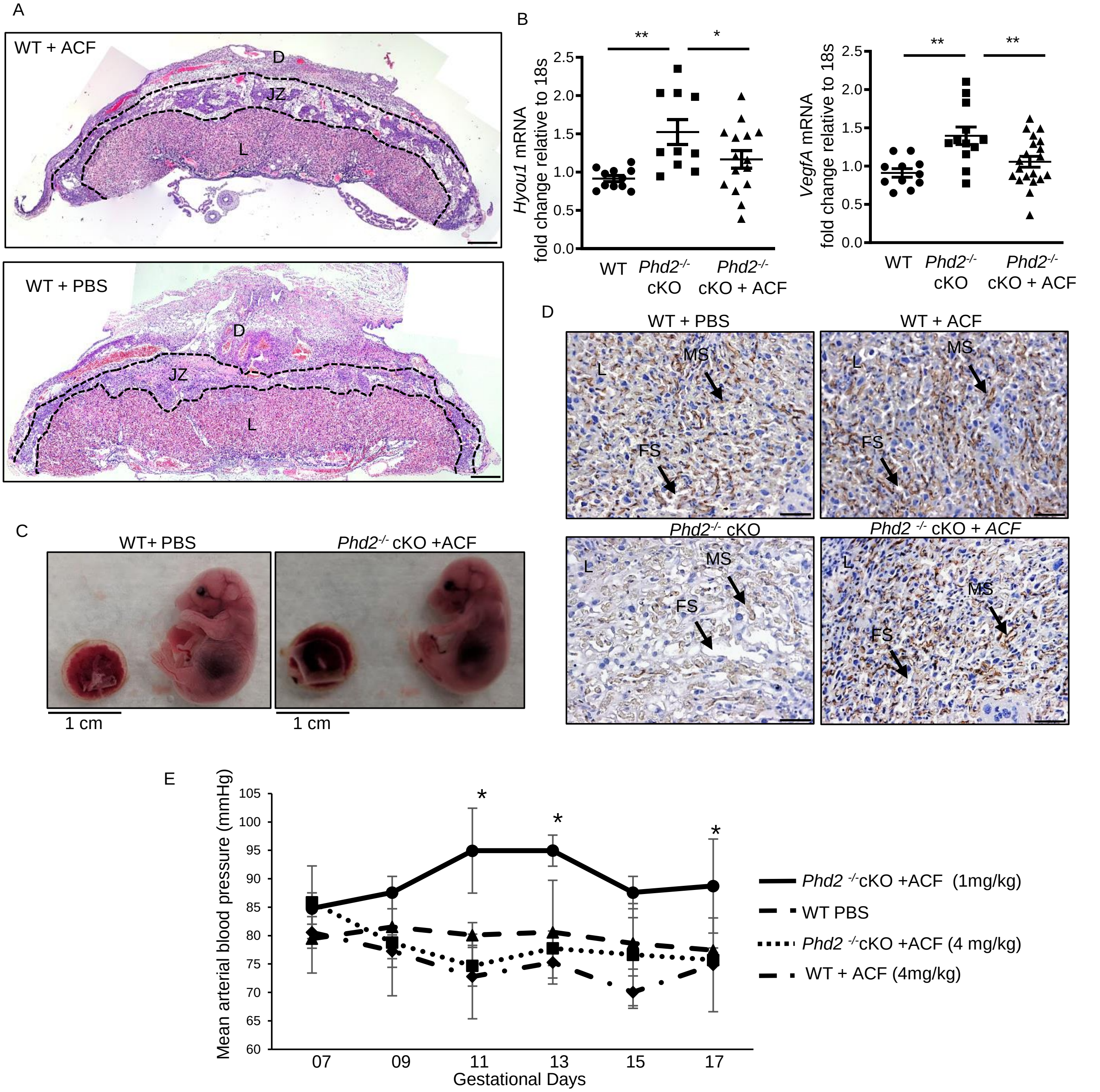

Fig. S5. (A) Representative H&E-stained whole placental section from WT mice treated with 2mg/kg of ACF or control vehicle PBS during early (GD7.5-14.5) pregnancy (magnification 10X, scale bars represent 300μm). D: decidua; JZ: junctional zone; L: labyrinth. **(B)** qPCR of *Hyou1* and *Vegfa* in E17.5 placentae from WT and $Phd2^{-/-}$ cKO pregnant mice treated with either PBS or ACF (*p<0.05, one-way ANOVA, Neuman-Keuls posthoc-test, n=13 WT placentae, n=14 $Phd2^{-/-}$cKO placentae, n=19 $Phd2^{-/-}$cKO +ACF placentae) **(C)** Gross morphology of E17.5 placentae and fetuses from WT and $Phd2^{-/-}$ cKO mice treated with either PBS or ACF during early (GD7.5-14.5) pregnancy. **(D)** Representative IHC staining for CD34 (endothelial cell marker) in E17.5 placentae from WT and $Phd2^{-/-}$cKO mice after treatment with PBS or ACF during early (GD7.5-14.5) pregnancy (magnification 20X, scale bars represent 50μm). L: labyrinth; FS: fetal sinusoids; MS: maternal sinusoids. **(E)** Mean arterial blood pressure across gestation in WT pregnant mice injected during early (GD7.5-14.5) pregnancy with either PBS or 4mg/kg ACF and $Phd2^{-/-}$ cKO pregnant mice treated with either 1mg/kg or 4 mg/kg ACF (*p< 0.05 relative to the WT pregnant mice, one-way ANOVA, Neuman-Keuls posthoc-test, n=3 separate pregnant mice per condition).

Full unedited gels for Figure 1 Panel C

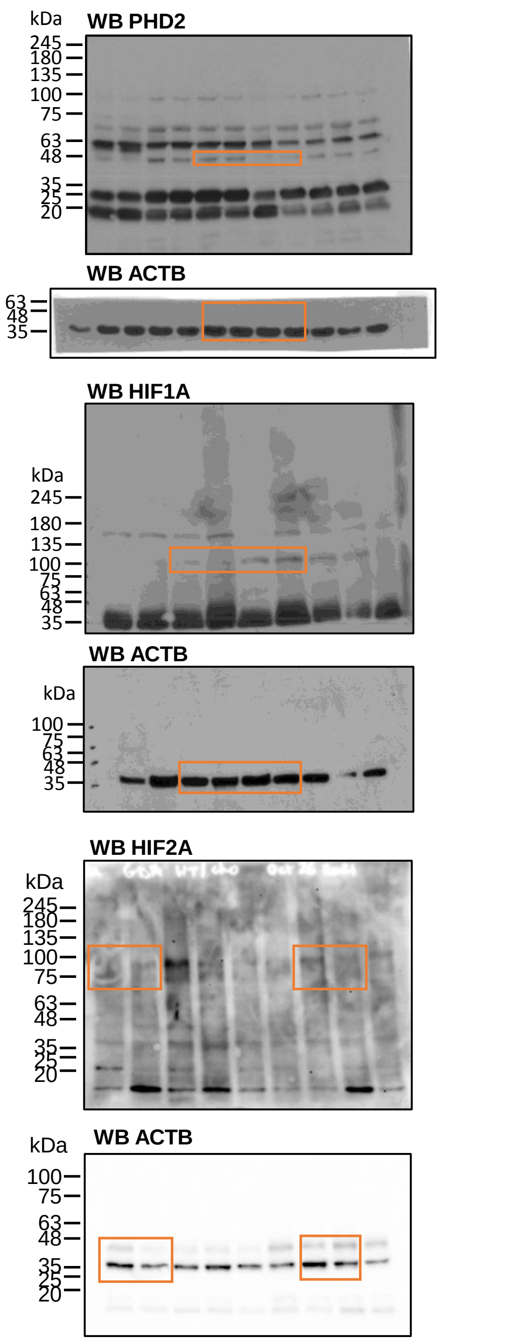

Full unedited gels for Figure 6 Panel C

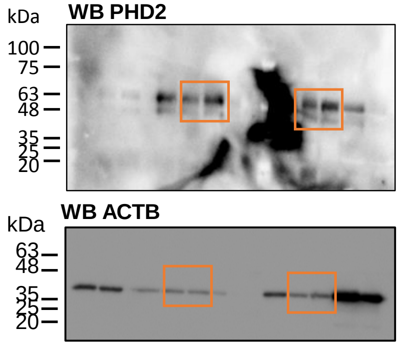

Full unedited gels for Figure S1 Panel A

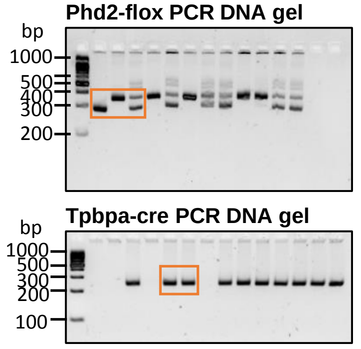

Full unedited gels for Fig S1 Panel D

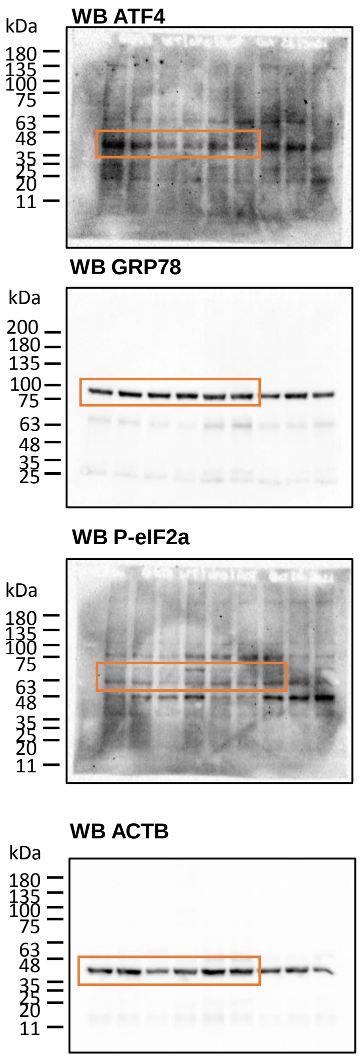

Supplement: Supplemental data [file jciinsight-7-158908-s120.pdf]
